# Whole-cell modeling predicts alternative proteome allocation strategies in the archaeon *Methanococcus maripaludis*

**DOI:** 10.1038/s41598-026-37887-z

**Published:** 2026-02-19

**Authors:** Ghada S. Kasem, Taysir Hassan A. Soliman, Mohamed A. Ali Mousa, Zeinhum F. Jaheen, Ibrahim E. Elsemman

**Affiliations:** 1 Department of Information Systems, Faculty of Computers and Information, Assiut University, Assiut, Egypt; 2 Department of Mathematics, Faculty of Science, Assiut University, Assiut, Egypt

**Keywords:** Biochemistry, Biotechnology, Computational biology and bioinformatics, Microbiology

## Abstract

The archaeon *Methanococcus maripaludis* (*M. maripaludis*) is a model organism for studying archaeal physiology and energy conservation in the hydrogenotrophic methanogenesis pathway. *M. maripaludis* has a distinct proteome allocation strategy, in which ribosomal proteome allocations do not change with growth rates. Here, we developed a proteome-constrained metabolic model that can explain this different proteome allocation strategy. First, we used multiple bioinformatics databases to compile information about the translational process and enzymatic complexes. We then extended a genome-scale metabolic model of *M. maripaludis* with protein synthesis processes, including ribosome assembly, tRNA charging, and enzyme assembly reactions. The proposed model predicts alternative proteome resource allocation strategies and mutant fitness for this archaeon under different conditions. Therefore, our model provides a framework for studying the effects of resource allocation on the hydrogenotrophic methanogenesis pathway.

**Supplementary Information:**

The online version contains supplementary material available at 10.1038/s41598-026-37887-z.

## Introduction

Genome-scale metabolic models (GEMs) comprise known biochemical reactions that illustrate how a studied organism uses nutrients to produce the required energy and biomass to grow under different conditions^1^. GEMs have been successfully used in various applications, such as metabolic engineering, human diseases, and microbial ecology^2–4^. The main limitations of GEMs are their ability to predict maximum growth rates and overflow metabolism, where cells shift from respiration to respiro-fermentative metabolism^1^. To address these limitations, the next generation of metabolic models (ME-models) was developed by extending GEMs with new biochemical reactions describing how the cell translates mRNA into peptides and how these peptides are assembled into ribosomes and metabolic enzymes^5^. These next-generation GEMs can predict maximum growth rates and overflow metabolism by adding new proteome constraints that limit protein abundance in GEMs^6,7^. Despite these advances, few next-generation GEMs have been constructed for a limited number of organisms, such as *Escherichia coli*^5^, *Saccharomyces cerevisiae*^8^, and *Lactococcus lactis*^9^. To the best of our knowledge, no next-generation GEMs for archaea have been developed to increase our physiological understanding of these important organisms^7^.

*M. maripaludis* is an anaerobic archaeon that can utilize either CO_2_ and H_2_ or formate as catabolic substrates to produce methane via the Wood–Ljungdahl pathway^10,11^. Studies on *M. maripaludis* have elucidated the role of hydrogen and energy conservation in this pathway^12–14^ and investigated how the organism can shift from growth to maintenance under low energy flux conditions^12,15,16^. Owing to these insights and the availability of genetic tools^17^, *M. maripaludis* is considered a model organism for studying archaeal physiology^18^. Furthermore, it has been employed in metabolic engineering as a microbial cell factory to produce bioplastics and various biochemical products, including hydrogen, methanol, geraniol, and amino acids^19–21^.

Four GEMs have been developed for *M. maripaludis*. The first model studied the mutualistic interaction between *M. maripaludis* and *D. vulgaris*. This model comprises 170 reactions and 147 metabolites^22^. The second model (iMM518) comprises 570 reactions, 556 metabolites, and 518 genes, covering 30% of its ORFs, focusing on core metabolism and enhancing methane production from CO₂^23^. The third model (iMR539) comprises 688 reactions, 710 metabolites, and 539 protein-encoding genes, covering 31% of the organism’s ORFs. The authors re-annotated and updated more reactions with enzymatic complexes in the hydrogenotrophic methanogenesis pathway, especially the central role of electron bifurcation, and biosynthesis of coenzymes^24^. Finally, the fourth model (iMR557) was refined to explore the impact of nitrogen fixation using N₂ on carbon conversion and methanogenesis. This model includes 716 reactions, 726 metabolites, and 557 protein-encoding genes (32% ORF coverage)^25^. The predictive power of these GEMs is their ability to estimate growth yields and methane fluxes. However, these models cannot estimate the cost of protein biosynthesis and how the proteins are allocated under low growth conditions^12^.

Müller et al.^12^ and Gu et al.^26^ generated two high-quality experimental proteomic data sets at different dilution rates under formate and phosphate limitations^12,26^. These studies revealed that the proteome allocation concept used by *M. maripaludis* differs from that of *E. coli* and budding yeast. Under formate-limited conditions, *M. maripaludis* produces a constant ribosomal proteome fraction at different growth rates, whereas *E. coli* and budding yeast produce ribosomal proteome fractions that increase with growth rates^8,27^. However, under phosphate-limited conditions, the ribosomal proteome fraction increases with the growth rate, similar to that observed in *E. coli* and yeast. Based on these studies, we hypothesized that these proteomics data sets can be used to estimate important parameters, such as the ribosomal turnover rate and the mass fraction of proteins other than enzymes and ribosomes, for the next-generation GEM of *M. maripaludis*. This new model would predict ribosomal proteome fractions at different growth rates.

In this study, we used the experimental data from Müller et al.^12^ and Gu et al.^26^ to reconstruct the first proteome-constrained model for *M. maripaludis* (pcMMP) by integrating proteome constraints into the iMR539 model^8^. The new model comprised 4,801 reactions, 2,483 metabolites, and 614 genes, covering 35% of the organism’s ORFs. The pcMMP model successfully predicted specific maximum growth rates, growth yields, and methane fluxes. This model predicted that ribosomal proteome fractions were constant with growth rates under formate-limited conditions. Therefore, this new model can be used in further studies to explore the methane production capabilities of *M. maripaludis* and guide metabolic engineering.

## Methods

### Collecting data

**Fig. 1:**
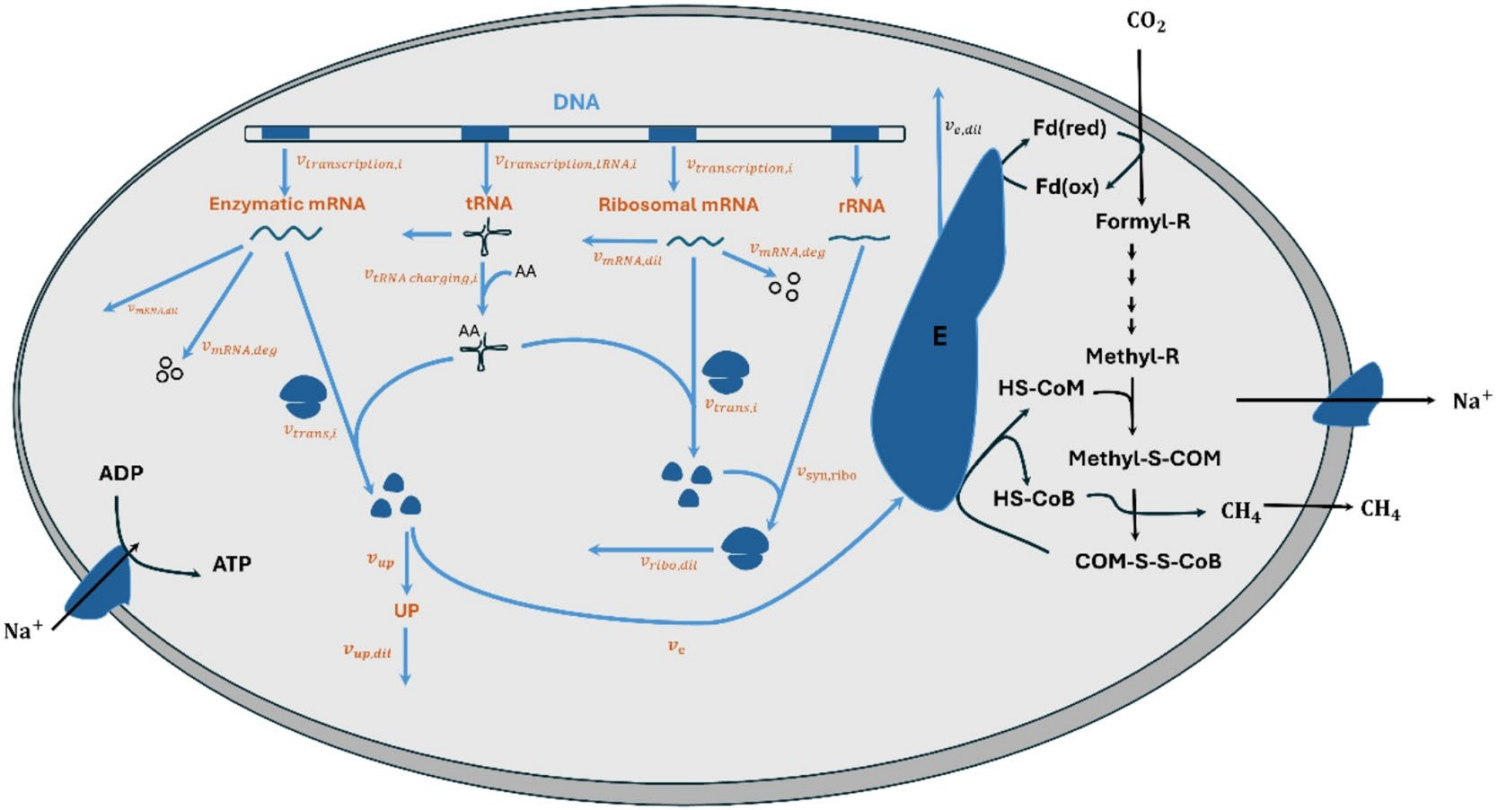
Overview of the pcMMP model. This figure was adapted from Halim et al.^18^. The black and blue reactions are from the iMR539 model and the new reactions in pcMMP, respectively. These new reactions allow the model to translate peptides and assemble them into ribosomes, enzymatic complexes, and the UP proteins. The new reactions also contain rRNA, tRNA, and mRNA transcriptional reactions. For each complex, we added a dilution reaction that determines the diluted abundances to the daughter cell. The model description is provided in the Supplementary Information file.

To reconstruct the proteome-constrained model of *M. maripaludis* (pcMMP), we updated the iMR539 model with new reactions describing the gene expression machinery in *M. maripaludis* (Fig. 1). We first retrieved the gene and protein sequences and annotations, including start and end positions, and strand direction from the National Center for Biotechnology Information (NCBI) database (taxonomy ID: 267377)^28^. Additionally, we retrieved tRNA gene data from the Genomic tRNA Database (GtRNAdb)^29,30^ and rRNA and ribosomal protein data from the KEGG database^31,32^. The translation factors were compiled from a previous study^10^.

We also compiled enzymatic turnover rates from the BRENDA database^33^, GotEnzymes^34^, and a literature review (Supplementary Data 1). The subunit compositions and stoichiometries of enzyme complexes were curated from the RCSB Protein Data Bank (PDB)^35^ (Supplementary Data 2). In the case of using the BRENDA or PDB database, values were collected first from *M. maripaludis*, second from related methanogenic archaea, and third from other archaeal organisms. For enzymes which have no archaeal turnover rates, we used the minimum bacterial turnover rates because the median bacterial turnover rate (100 per second)^9^ was higher than that for archaea^34^. The complexes with no structural information were assumed to be monomers. The molecular weights of peptides were calculated using the PyOpenMS package^36^.

Furthermore, we fixed the median of the collected turnover rates (5 per second) for enzymes lacking a turnover rate^9^. The use of small turnover rates allows the model to produce a larger proteome fraction for these enzymes than the measured fraction. Therefore, we fixed the rate to 0.75 per second for each enzyme with a turnover rate below 0.75 per second. We estimated the proteome fractions for enzymes with a default turnover rate of 5 or 0.75 per second (Supplementary Fig. S1). The difference between the predicted and measured proteome fractions was approximately 6% of the total proteome mass. This difference was also included in the UP cost ratio and did not affect our simulations.

### Network reconstruction of the PcMMP model

We used the COBRApy^37^ package in Python to reconstruct the iMR539 GEM of *M. maripaludis* to the pcMMP model. The reversible reactions were divided into two irreversible reactions (Supplementary Data 3). Reactions associated with isozymes (which were identified by logical “or” operators in their Gene-Protein-Reaction associations) were separated into distinct reactions, where each reaction was associated with a unique isozyme (Supplementary Information and Supplementary Data 3). Subsequently, we compiled a list of template reactions representing gene expression processes, including translation, transcription, tRNA charging, mRNA degradation, ribosome assembly, complex formation, and dilution reactions (Supplementary Information).

The biomass reaction requires an amount of ATP (GAM) to produce one gDW of protein, RNA, DNA, lipids, and carbohydrates^38^. As the pcMMP model consumed an amount of ATP molecules to produce protein and RNA, we removed the protein and RNA compositions from the biomass reaction and changed the GAM value. We found that the mass of the other compositions was 0.07 gDW (Supplementary Table S1). These compositions were lipid species and other vitamins. Müller et al.^12^ found that the cell diameter did not change at different growth rates. Therefore, we assumed that there was no change in the lipid composition, and we kept the mass of the remaining compositions as 0.07 gDW.

Finally, we added GAM and Unspecific Protein (UP) reactions. The final reconstruction (4,801 reactions) was exported to Systems Biology Markup Language (SBML).

### Main constraints of the PcMMP model

To simulate the pcMMP model, we added the enzyme capacity constraint (coupling between metabolic fluxes and enzyme abundances) and the ribosome capacity constraint (coupling between protein and ribosome abundances). Supplementary Information contains additional details about the model description. For each growth rate (µ), we created a function that solved a linear programming problem using the Soplex solver^39^. The core linear programming problem is defined as follows:

Minimize: *v*_*uptake*_.

Subject to.

1. The mass balance constraints:

$$\:S.v=0$$

This constraint is used in flux balance analysis, where *S* is the stoichiometric matrix and $\:v$ is the vector of all fluxes in the model.

2. Enzyme capacity constraints:

$$\:{v}_{i\:}-\frac{{k}_{cat}}{{\upmu\:}}\:.\:{v}_{e}=0$$

where $\:{v}_{i\:}$is the flux value of the *i*^*th*^ reaction in the model, while $\:{v}_{e}$ is the formation flux of the metabolic complex *e* that carries out the *i*^*th*^ reaction.

3. Ribosome capacity constraints:

$$\:{v}_{trans,i\:}-\frac{{k}_{cat,ribo}}{\mathrm{l}\mathrm{e}\mathrm{n}\mathrm{g}\mathrm{t}\mathrm{h}\left({\mathrm{p}\mathrm{e}\mathrm{p}\mathrm{t}\mathrm{i}\mathrm{d}\mathrm{e}}_{i}\right)}\:.\:{eR}_{i}=0$$

$$\:\sum\:{eR}_{i}-\frac{1}{{\upmu\:}}\:.\:{v}_{syn,ribo}\:=0$$

The first constraint couples the flux ($\:{v}_{trans,i\:}$) of a translated peptide *i* with the required ribosomal fraction ($\:{eR}_{i}$), where $\:{k}_{cat,ribo}$ is the ribosomal turnover rate. The second constraint couples the ribosome assembly flux ($\:{v}_{syn,ribo}$) with the summation of the required ribosomal fractions in the model.

4. UP constraint:

$$\:UP\ge\:c$$

This constraint enforces the model to produce a specific amount (*c*) of UP that is not included in the model. The *c-*value is estimated under different conditions.

5. Coupling mRNA constraints:

$$\:{v}_{trans,i\:}-\frac{{k}_{mRNA}}{{k}_{deg}+\mu\:\:}\:.\:{v}_{transcription,i}=0$$

This constraint couples $\:{v}_{trans,i}$ and the mRNA transcriptional flux ($\:{v}_{transcription,i\:}$), where $\:{k}_{mRNA}$ and $\:{k}_{deg}$ are catalytic and degradation rates of mRNA, respectively.

6. Coupling tRNA constraints:

$$\:{v}_{tRNA\:charging,i\:}-\frac{{k}_{tRNA}}{\mu\:\:}\:.\:{v}_{transcription,tRNA,i}=0$$

This constraint couples the flux of tRNA charging ($\:{v}_{tRNA\:charging,i\:})\:$with the flux of tRNA transcription ($\:{v}_{transcription,tRNA,i}$), where $\:{k}_{tRNA}$is the tRNA catalytic rate.

7. The total proteome constraint:

$$\:\sum\:\frac{{v}_{e}}{\mu\:}\:.\frac{{mw}_{e}}{1000}+\left(\frac{{v}_{syn,ribo}}{\mu\:}\:.\frac{{mw}_{r-proteins}}{1000}\right)+\left(\frac{{v}_{UP}}{\mu\:}\:.\frac{{mw}_{UP}}{1000}\:\right)-Total\:Protein=0$$

8. The total RNA mass constraint:

$$\:\frac{{v}_{syn,ribo}}{\mu\:}\:.\frac{{mw}_{rRNA}}{1000}-rRNA=0$$

$$\:\sum\:\frac{{v}_{transcription,i}}{\mu\:+{k}_{deg}}\:.\frac{{mw}_{{mRNA}_{i}}}{1000}-mRNA=0$$

$$\:\sum\:\frac{{v}_{transcription,tRNA,i}}{\mu\:}\:.\frac{{mw}_{{tRNA}_{i}}}{1000}-\:tRNA=0$$

$$\:rRNA+mRNA+tRNA-RNA=0$$

9. The glycogen mass constraint:

$$\:\frac{{v}_{Glycogen}}{\mu\:}\:.\frac{{mw}_{Glycogen}}{1000}-Glycogen=0$$

10. The macromolecular composition constraint:

$$\:Total\:Protein+RNA+Glycogen=0.93$$

### Model validation

To validate our model, we used high-quality chemostat experiments for *M. maripaludis* under formate- and phosphate-limited conditions^12,26^. Methane fluxes, growth yields, and proteome mass fractions were also measured at each dilution rate. Gu et al.^26^ measured methane fluxes in units of µmol OD^− 1^mL^− 1^ h^− 1^. To convert this unit to mmol gDW^− 1^ h^− 1^, we divided the measured fluxes by the dry weight conversion factor (0.34 gDW L^− 1^)^12,24^. Gu et al.^26^ measured the protein and RNA masses in units of µg OD^− 1^ mL^− 1^. To convert this unit to grams of protein or RNA per gDW, we divided each measured mass by 340.

For chemostat data sets under CO_2_-limited conditions, we used the growth yields and methane fluxes from Richards et al.^24^. Finally, to assess the model’s ability to predict the fitness of hydrogenase-deficient mutants (Δ3H₂ase, Δ5H₂ase, Δ6H₂ase, ∆6H_2_ase-∆*cdh*, and suppressor strains: Δ6H₂ase_sup_, Δ7H₂ase_sup_), we used experimental data from Li et al.^14^ and Costa et al.^13^.

### Estimation of ribosomal turnover rates

The ribosomal turnover rate ($\:{k}_{cat,ribo}$) is a key parameter in the model. To estimate $\:{k}_{cat,ribo}\:$at a certain growth rate ($\:\mu\:$), we applied the following equation from the ME-model of *E. coli*^5^ and the pc-model of *L. lactis*^9^ as follows:

1 $$k_{{cat,ribo}} = ~\mu ~.~\frac{{~P/m_{{aa}} }}{{R~.~f_{{rRNA}} /m_{{rr}} }}$$

Equation (1) estimates the number of amino acids and ribosomes per gDW. The number of amino acids is estimated by dividing the protein mass (*P*) by the average molecular weight of an amino acid ($\:{m}_{aa})$. The number of ribosomes per gDW is estimated by dividing the rRNA mass by the molecular weight of rRNA ($\:{m}_{rr})$, where rRNA is a fraction ($\:{f}_{rRNA})$ from the total RNA mass. Therefore, the parameter $\:AA\:per\:ribosome$ is defined as follows:

2 $$AA~per~ribosome = \frac{{~P/m_{{aa}} }}{{R~.~f_{{rRNA}} /m_{{rr}} }}$$

Therefore, Eq. (1) can be rewritten as follows:

3 $${k}_{cat,ribo}=\:\mu\:\:.\:AA\:per\:ribosome$$

To estimate the $\:{k}_{cat,ribo}$ value under formate-limited conditions, we used the measured masses of protein (0.6 g/gDW) and RNA (0.14 g/ gDW) at a specific maximum growth rate (0.23 per hour), where the translation rate reached a maximum value at high growth rates^40^. Using these measurements, the value of $\:AA\:per\:ribosome$ was estimated to be 19.8 × 3600 (aa/ribosome). However, when we used this value, the model predicted ribosome abundances that were higher than that measured (Supplementary Fig. S2). We adjusted the value of $\:AA\:per\:ribosome$ to be 3600 × 22 (aa/ribosome). Therefore, $\:{k}_{cat,ribo}$ became $\:3600\:\times\:\:22\:\mu\:\:$(aa/hour), and this value was used in all simulations, except for the phosphate-limited conditions.

Under phosphate-limited conditions, Gu et al.^26^ reported that the ribosomal proteome fraction was lower than that under formate-limited conditions at low growth rates. The polysome fractions were also higher than those under formate-limited conditions. The minimum measured polysome fractions at a growth rate of 0.003 per hour were 10% and 18% under formate- and phosphate-limited conditions, respectively^26^. Therefore, the ribosomal turnover rate could be increased 1.8-fold under phosphate-limited conditions. Additionally, the ribosomal proteome fractions were equal under phosphate- and formate-limited conditions at a growth rate of 0.1 per hour. These data indicate that the difference in ribosomal turnover rates decreases with the growth rate.

We therefore changed $\:AA\:per\:ribosome$ values until the model could estimate the measured ribosomal proteome fraction at a growth rate of 0.002 per hour. We estimated the value of $\:AA\:per\:ribosome$ as 3600 × 40 (aa/ribosome). This fold change in the $\:AA\:per\:ribosome$ was consistent with the measured polysome fraction. Finally, we drew the regression line between the points (0.002,40) and (0.1,22) (Supplementary Fig. S3a) to estimate $\:{k}_{cat,ribo}$ at each growth rate under phosphate-limited conditions (Supplementary Fig. S3b). In this case, the model could predict the increase in ribosome abundances under phosphate-limited conditions.

### Simulation

For chemostat simulations, we adjusted the growth medium at each dilution rate between 0.002 and 0.1 per hour (Supplementary Table S2). We also minimized the formate and phosphate uptake rates under formate- and phosphate-limited conditions, respectively^41^. The model could use formate as the electron donor and sole carbon source but could not use hydrogen^12^. Finally, the model could take up protons because the absence of protons from the medium affects the growth of *M. maripaludis*^24^.

For the batch simulation, we searched for the specific maximum value ($\:{\mu\:}_{max}$) of the parameter $\:\mu\:$ in the model formula such that the LP problem became infeasible above $\:{\mu\:}_{max}$. The value of $\:{\mu\:}_{max}$ represents the predicted specific maximum growth rate^5,8,9^. To this end, we used a binary search algorithm to find $\:{\mu\:}_{max}$ between the values 0.0 and 0.5 per hour. The binary search started at a specific growth rate of 0.25 per hour and solved the LP problem. If a feasible solution exists for this problem, the binary search continues the search in the upper bound (between 0.25 and 0.5 per hour). Otherwise, the binary search continues the search in the lower bound (between 0.0 and 0.25 per hour). Therefore, this approach efficiently narrowed the search space until the specific maximum growth rate was found.

We used the wild-type strain MM901 to simulate knockout studies. The Δ3H₂ase mutant was simulated by deactivating the genes *frcA*, *frcG*, *frcB*, *fruA*, *fruG*, *fruB*, and *hmd*. The Δ5H₂ase mutant included the deactivation of *fruA*, *frcA*, *hmd*, *vhuA*, *vhuU*, and *vhcA*. The Δ6H₂ase mutant was constructed by extending the Δ5H₂ase mutant with *ehbN* deactivation. In the ∆6H_2_ase-∆*cdh* mutant, we extended the ∆6H_2_ase mutant by deactivating *cdh.* To reflect the experimental data, the glyceraldehyde-3-phosphate ferredoxin oxidoreductase (GAPOR) reaction was constrained to zero flux in all mutants and was only reactivated in the suppressor mutants (Δ6H₂ase_sup_ and Δ7H₂ase_sup_). The Δ7H₂ase_sup_ mutant incorporated the deactivation of *ehaN* and *ehaO* from the Δ6H₂ase_sup_ mutant.

To simulate knockout mutants, we first predicted $\:{\mu\:}_{max}\:$for the wild type grown on formate as the electron donor and sole carbon source, with acetate supplementation in the growth medium and in the absence of hydrogen. Then, we set the flux of reactions that translate the knockout proteins to zero. Finally, we used the binary search algorithm to predict a specific maximum growth ($\:{{\upmu\:}}_{max}^{mutant}$) for each mutant.

To estimate the measured wild type and mutant fitness, we used the online toolbox PlotDigitizer to extract the data points from the growth curves at the beginning and end of the log phase (Supplementary Table S3). The growth rate (*r*) can be estimated as follows:

$$\:r=\:\frac{\mathrm{ln}\left({OD}_{2}\right)-\:\mathrm{ln}\left({OD}_{1}\right)\:}{{t}_{2}-\:{t}_{1}}$$

Finally, the predicted and measured fitness values were estimated as follows:

$$\:fitness=\:\frac{{{\upmu\:}}_{max}^{mutant}}{{\mu\:}_{max}}$$

## Results and discussion

### Description of the PcMMP model

We reconstructed the pcMMP model by extending the iMR539 model^24^ with new reactions and genes representing the gene expression machinery. The pcMMP model comprises 4,801 reactions, 2,483 metabolites, and 615 genes, including the UP gene (Table 1). The model contains 1,322 metabolic reactions and 615 translational reactions, 1,845 reactions related to mRNA processing (transcription, degradation, and dilution), 186 reactions related to tRNA processing (transcription, charging, and dilution), and 20 reactions related to rRNA processing. Furthermore, the pcMMP model includes 810 metabolic complex reactions (formation and dilution), two reactions for ribosome formation and dilution, and one reaction for UP dilution.

In the pcYeast model^8^, the biomass compositions were changed at each growth rate, and the protein mass was fixed based on the measured data. Moreover, the pcLactis model constrained the protein mass to a specific value^9^. We added the following biomass constraint:

4 $$Total\:Protein+RNA+Glycogen=0.93$$

Equation (4) constrains the model to produce a total mass of protein, RNA, and glycogen equal to 0.93 gDW. The model can produce a small amount of protein mass at low growth rates. The exact biomass compositions must be defined to satisfy this mass constraint. Because the biomass compositions were not available at these low growth rates and under phosphate-limited conditions, we used another approach by allowing the model to increase the glycogen mass. This approach was consistent with the biomass compositions (protein, RNA, and glycogen) reported by Sher et al.^42^. An increase in the predicted glycogen mass refers to the existence of other biomass compositions. Therefore, the proposed approach avoided the addition of new constraints that describe the biomass compositions before simulations.

**Table 1:** Description of the PcMMP model.

Process	Number of reactions	Number of proteins
Total	4801	615
Metabolic network	1322	540
Translation processing	615	75
RNA processing	2051	0
Formation and dilution of the ribosome and metabolic complexes, and the UP protein.	813	0

### Estimation of the UP cost ratio

The pcMMP model estimated the minimum protein mass required for growth. Consequently, the predicted protein mass may be lower than the measured protein mass. We increased the UP cost ratio to reflect the biological cost under formate-limited conditions. This was consistent with the pcYeast model, in which the UP cost ratio was changed under different carbon sources^8^.

We used a proteomic data set at a specific maximum growth rate (0.23 per hour) to estimate the UP cost ratio. The cost of the unmodeled proteome mass fractions that were not included in the model was 44% of the total protein. Under formate-limited conditions, the measured protein mass and metabolic proteome allocation were 20% and 6% higher than the predicted values, respectively (Supplementary Fig. S4a). Therefore, the UP cost ratio was adjusted to 75% of the total protein mass to reach the measured protein mass of 0.7 g/gDW (Table 2 and Supplementary Fig. S4a). These results indicate that *M. maripaludis* can produce more proteins that are not required for growth.

Under phosphate-limited conditions, we used the UP cost ratio of 44% of the total protein mass (Table 2). The predicted protein mass was consistent with the measured data (Supplementary Fig. S4b). These results indicate that *M. maripaludis* can produce the proteins required for growth.

*M. maripaludis* had different methane fluxes under different growth conditions at growth rates between 0.002 and 0.1 per hour. The GAM value has a significant impact on methane fluxes (Supplementary Fig. S5). Therefore, we used GAM values that match the methane fluxes (Table 2). For NGAM values, Müller et al.^12^ estimated that the NGAM value under formate-limited conditions was between 0.51 and 1.61 mmol ATP gDW^− 1^ h^− 1^. We used the NGAM value of 1 mmol ATP gDW^− 1^ h^− 1^ under formate- and phosphate-limited conditions. Under CO_2_-limited conditions, we used the NGAM value from the iMR539 model^24^ as 5 mmol ATP gDW^− 1^ h^− 1^.

**Table 2:** Estimation of the UP cost ratio used in our simulation.

Conditions	Estimated UP cost ratio	Saturation factor	Differences in protein mass	The UP cost ratio used in the model	GAM (mmol ATP gDW^− 1^)	NGAM (mmol ATP gDW^− 1^ h^− 1^)
Chemostat (Formate)	44%	6%	20%	75%	25	1
Chemostat (Phosphate)	44%	0	0	44%	70	1
Batch (Formate)	44%	0	0	44%	25	1
Chemostat (CO_2_ and H_2_)	44%	0	0	44%	130	5
Batch (CO_2_ and H_2_)	20%	0	0	20%	25	5

### pcMMP predicts protein and RNA masses, and growth yield in *M. maripaludis*

**Fig. 2:**
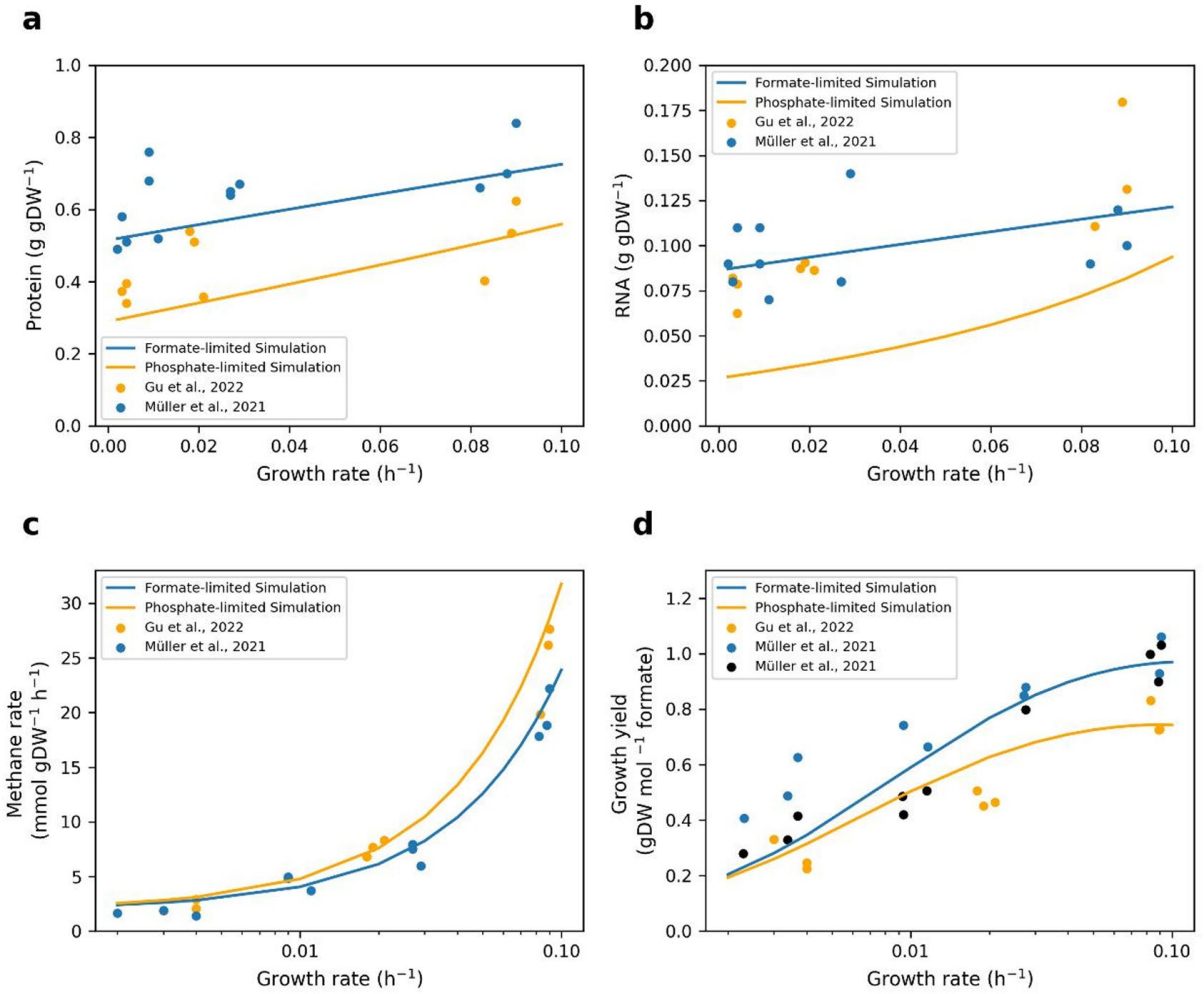
Predicted and measured response of *M. maripaludis* to growth under formate- and phosphate-limited conditions. The lines represent the model predictions, and the circles represent the measured data^12,26^. The blue and orange colors represent the formate- and phosphate-limited conditions, respectively. (**a**) Comparison of the predicted ratio of protein mass (g protein/gDW) with the measured data. (**b**) Comparison of the predicted ratio of RNA mass (g RNA /gDW) with the measured data. (**c**) Comparison of the predicted methane flux (mmol gDW^−1^ h^−1^) with the measured flux. (**d**) Comparison of the predicted yield (gDW mol^−1^ formate) with the measured yield. Under formate-limited conditions, the black and blue symbols refer to the observed growth yield and the corrected growth yield with cell lysis, respectively. Orange symbols indicate the growth yield under phosphate-limited conditions.

We simulated the model under formate- and phosphate-limited conditions. Interestingly, the model could predict the protein and RNA masses (Fig. 2a and b). The predicted protein and RNA masses were aligned with the minimum measured masses. For example, under formate-limited conditions, at low growth rates (between 0.002 and 0.01 per hour), the measured protein mass was between 0.49 and 0.76 g protein per gDW, and the RNA mass was between 0.07 and 0.11 g RNA per gDW. The predicted protein mass was between 0.51 and 0.53 g protein per gDW, and the predicted RNA mass was between 0.087 and 0.089 g RNA per gDW.

At low growth rates, the model predicted a high glycogen mass and low protein and RNA masses. The model required more enzymes, ribosomes, and RNA as the growth rate increased. Consequently, the glycogen mass decreased with growth rate (Supplementary Fig. S6). Under phosphate-limited conditions, the predicted RNA mass was lower than the measured data because the model produced the minimum ribosome abundances required for growth. As rRNA is the majority mass of RNA, *M. maripaludis* may produce more rRNA that is not assembled into ribosomes. This result was observed in the measured data. The measured RNA masses were similar under phosphate- and formate-limited conditions, whereas the ribosomal proteome fraction was lower under phosphate-limited conditions than under formate-limited conditions. Additionally, the model predicted methane fluxes under formate- and phosphate-limited conditions, which were compared with measured data (Fig. 2c).

At low growth rates, the model predicted lower growth yields than the measured data. Müller et al.^12^ gave two reasons for the low yield under formate-limited conditions: cell lysis and maintenance energy (ATP yield of 0.5 mol ATP per mol methane). In addition, they reported a nonlinear relationship between maintenance energy requirements and growth rates. However, our simulations considered a constant GAM value, whereas the measured data suggest that GAM varies under different growth conditions to optimize yield (Fig. 2d).

**Fig. 3:**
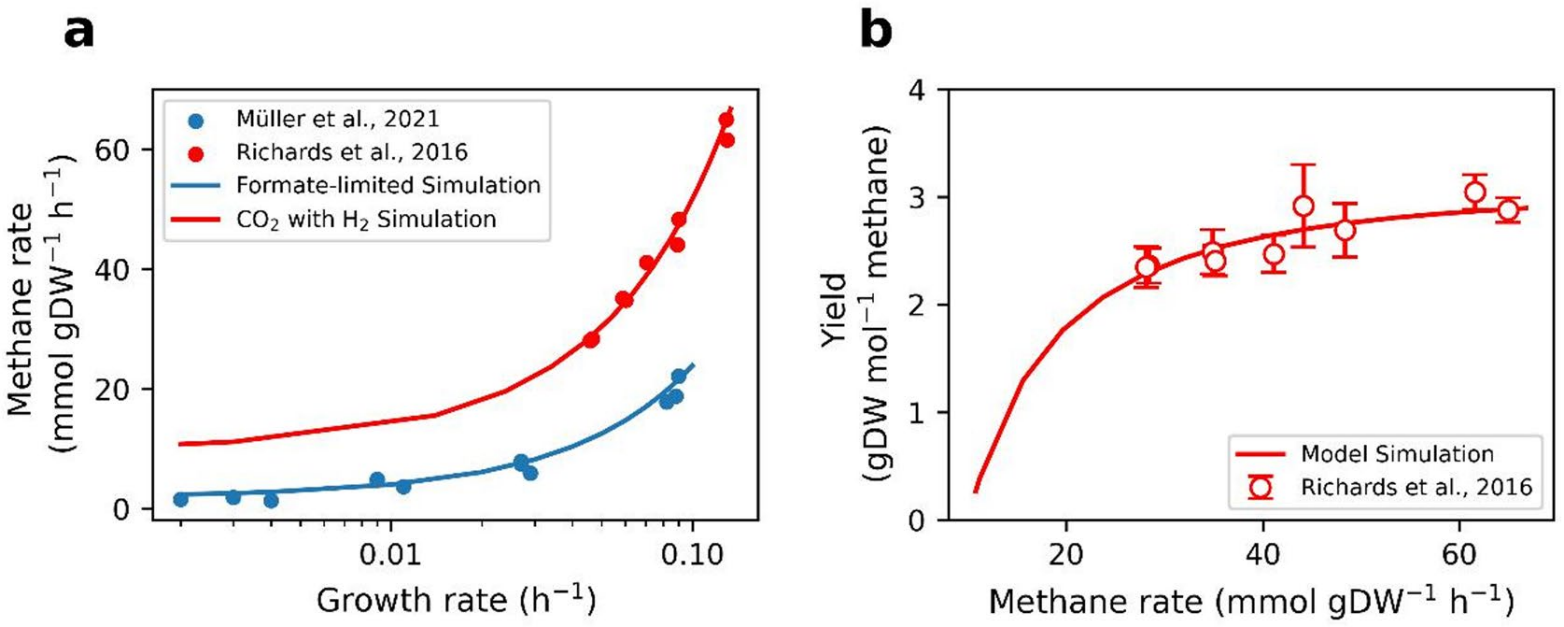
Predicted and measured methane fluxes and yield under CO _2_ -limited conditions at different growth rates. The lines represent the model predictions, and the symbols represent the measured data^12,24^. The red and blue colors represent CO_2_- and formate-limited conditions, respectively. (**a**) Measured methane fluxes (mmol gDW^−1^ h^−1^) under the CO_2_- and formate-limited conditions. The methane fluxes are higher under CO_2_-limited conditions than under formate-limited conditions. Therefore, we used higher GAM and NGAM under CO_2_-limited conditions. (**b**) Comparison of predicted yield (gDW mol^−1^ methane) with the measured yield under CO_2_-limited conditions^24^.

To simulate the pcMMP model under CO₂-limited conditions, we allowed the model to take up CO₂ as the sole carbon source, hydrogen as the electron donor, and acetate^24^. We also fixed the GAM and NGAM values in our simulations. The measured methane fluxes under CO₂-limited conditions were higher than those under formate-limited conditions (Fig. 3a). Therefore, we increased the GAM and NGAM to 130 mmol ATP gDW^− 1^ and 5 mmol ATP gDW^− 1^ h^− 1^, respectively. The model predictions were consistent with the measured data reported by Richards et al.^24^ (Fig. 3a and b).

### pcMMP predicts proteome allocation in *M. maripaludis*

**Fig. 4:**
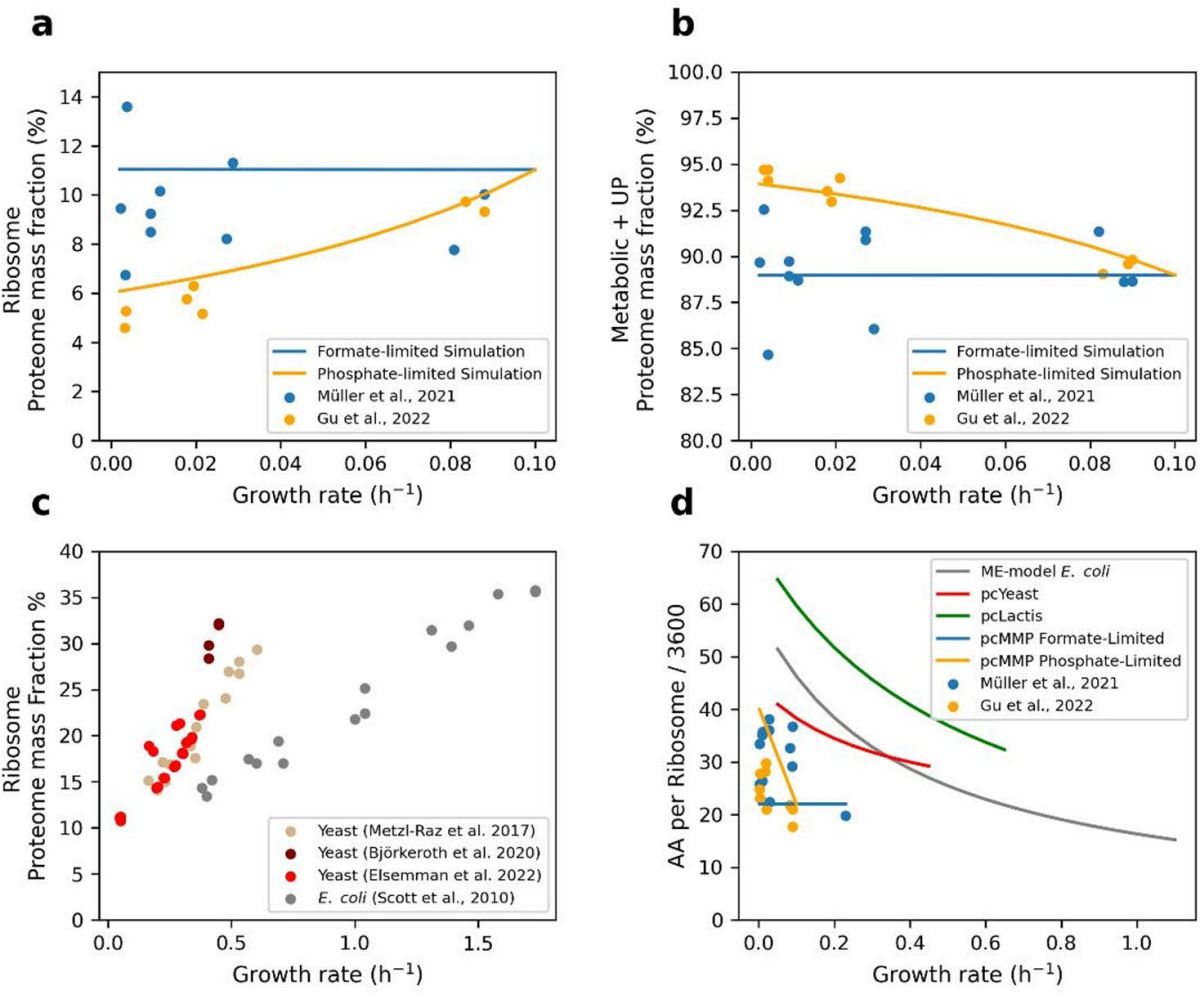
Comparison of the predicted minimal proteome fractions required for growth under formate- and phosphate-limited conditions at different growth rates. The lines represent the model predictions, and the symbols represent the measured data^12,26^. The blue and orange colors are the formate- and phosphate-limited conditions, respectively. (**a**) Comparison of the predicted minimal ribosomal proteome fraction with the measured proteome fractions. (**b**) Comparison of the summation of the predicted minimal metabolic and UP proteome fractions with the measured proteome fractions. (**c**) Experimental ribosomal proteome fractions increase with growth rates in *E. coli*^27^ and budding yeast^8,43,44^. (**d**) Comparison of $\:AA\:per\:ribosome$ in the ME-model of *E. coli* and pc-models of pcLactis, pcYeast, and pcMMP.

We computed the masses of ribosomal and non-ribosomal (metabolic + UP) proteomes from the linear programming solutions. These proteome fractions were consistent with the measured proteome allocations under phosphate-limited conditions (Fig. 4a and b). To demonstrate that the UP cost ratio had no role in the constant ribosomal proteome fraction, we simulated the model with two UP cost values: 44% and 75% of the total protein (Supplementary Fig. S7). We found no change in the ribosomal proteome fraction. These predictions indicate that the abundance of ribosomes was not regulated under formate-limited conditions, as reported by Müller et al.^12^.

On the other hand, Gu et al.^26^ reported that *M. maripaludis* regulated ribosomes under phosphate-limited conditions. To simulate the model under these conditions, we found that the ribosomal proteome fraction did not change as under formate-limited conditions (Supplementary Figs. S8 and S9). The model predicted a higher ribosomal proteome fraction than the measured fraction at low growth rates. This means that the ribosomal turnover rate used in the model was lower than the experimental turnover rate. The difference between these rates could be related to higher polysome fractions under phosphate-limited conditions. To study the additional effect of an increase in polysome fractions, we increased the value of $\:{k}_{cat,ribo}\:$at different growth rates (Supplementary Fig. S3b). Therefore, the model could predict the increase in the ribosomal proteome fraction with growth rate (Fig. 4a and b), as reported by Gu et al.^26^. As the ribosomal fraction increased with the growth rate, the sum of metabolic and UP proteome fractions, and other biomass compositions decreased with the growth rate (Supplementary Fig. S10).

The model predicted different proteome allocations in *M. maripaludis* than in *E. coli*^27^ and budding yeast^8^. In *E. coli* and budding yeast, the ribosomal proteome fraction increases with growth rate (Fig. 4c). Interestingly, we found that the parameter $\:{k}_{cat,ribo}$ plays a key role in limiting the abundance of ribosomes in the model, especially the value of the parameter $\:AA\:per\:ribosome$. Therefore, we compared the values for the parameter of $\:AA\:per\:ribosome$ from the pcMMP model with the other values from the ME-model of *E. coli* and pc-models of *L. lactis* and budding yeast (Fig. 4d). This comparison showed that the value of $\:AA\:per\:ribosome$ decreased with the growth rate in the other models. For the pcMMP model simulated under formate-limited conditions, the value of $\:AA\:per\:ribosome$ was constant at different growth rates.

Furthermore, we compared the predicted minimum required proteome masses of the pathways: methanogenesis and ATP synthesis, which covers 24 ± 3%^12^ of the total protein mass. At a growth rate of 0.089 per hour, the model predicted a methanogenesis proteome mass that was 4% lower than the measured mass under formate-limited conditions (Supplementary Fig. S11). The difference in the proteome fraction was included in the UP cost ratio. However, the methanogenesis proteome fraction was consistent with that measured under phosphate-limited conditions. The predicted ATP synthesis proteome mass was also consistent with that measured under both formate- and phosphate-limited conditions (Supplementary Fig. S11).

In conclusion, these results demonstrate consistency between the model prediction and the measured biomass compositions, methane fluxes, growth yields, and ribosomal proteome fractions.

### The biological meaning of the predicted glycogen mass

Under formate-limited conditions, the model predicted that the glycogen mass decreased from ~ 0.3 gDW at the lowest growth rate to ~ 0.07 gDW at the highest growth rate (Supplementary Fig. S6). *Methanococcus thermolithotrophicus* produced a glycogen mass of 0.146 g per g protein^45^. If the protein mass was 0.6 gDW, our prediction may have a biological meaning.

*M. maripaludis* can produce a small amount of glycogen (0.02 g per gDW)^46^. The addition of this constraint (glycogen mass = 0.02) to the model allows for more production of proteins and RNA. These new predictions were also consistent with the measured data under formate-limited conditions (Supplementary Fig. S12). The model predicted protein and RNA masses of 0.78 and 0.128 g per gDW, respectively. Thus, the model satisfied the constraint in Eq. (4) by producing 0.91 gDW of protein and RNA. The model has equality constraints for enzyme and ribosome capacity, and the inequality (greater than or equal) constraint for producing the UP protein. Therefore, the model produced a higher UP cost ratio than the lower value (75% of the total protein). To validate the non-effect of the UP cost ratio on this simulation, we simulated the model with the UP cost ratio of 44% of the total protein. The model predictions did not change as the UP cost ratio decreased (Supplementary Fig. S12). This result indicates that the model satisfied the mass constraint in Eq. (4) by producing the UP protein. Therefore, the minimum number of constraints in the model can be used to understand the role of new constraints in model predictions.

Under phosphate-limited conditions, the model predicted that the glycogen mass decreased from ~ 0.6 gDW at the lowest growth rate to ~ 0.27 gDW at the highest growth rate (Supplementary Fig. S6). Gu et al.^26^ reported that phosphate-limited conditions were related to decoupling of catabolism from biomass formation. This means that instead of glycogen, cells can biosynthesize free amino acids and nucleotides. As we simulated the model under phosphate-limited conditions, we assumed that the model did not produce free nucleotides that require phosphate. Therefore, for simplicity, we assumed that cells produced only free amino acids. This can be modeled by defining the free amino molecule based on the amino acid compositions in the iMR539 metabolic model. Supplementary Table S4 shows the fraction of each amino acid in the free amino acid molecule. Finally, we modified the mass constraint as follows:

$$\:Total\:Protein+\:RNA\:+\:\mathrm{F}\mathrm{r}\mathrm{e}\mathrm{e}\:\mathrm{A}\mathrm{m}\mathrm{i}\mathrm{n}\mathrm{o}\:\mathrm{A}\mathrm{c}\mathrm{i}\mathrm{d}\:\mathrm{m}\mathrm{o}\mathrm{l}\mathrm{e}\mathrm{c}\mathrm{u}\mathrm{l}\mathrm{e}\:=0.93\:\:\:\:\:\:$$

In this case, the model filled this mass constraint with a free amino acid molecule mass instead of glycogen mass, and the model predictions were consistent with the experimental data under phosphate-limited conditions (Supplementary Figs. S13 and S14).

These results indicate that the predicted glycogen mass has different biological meanings under different conditions. Under formate-limited conditions, the predicted glycogen mass corresponded to the increase in protein mass, which was consistent with the measured data. Under phosphate-limited conditions, the predicted glycogen mass referred to the free amino acid mass, which was also consistent with the concept of decoupling of catabolism from biomass formation^26^.

### pcMMP predicts maximum growth rates of *M. maripaludis*

**Fig. 5:**
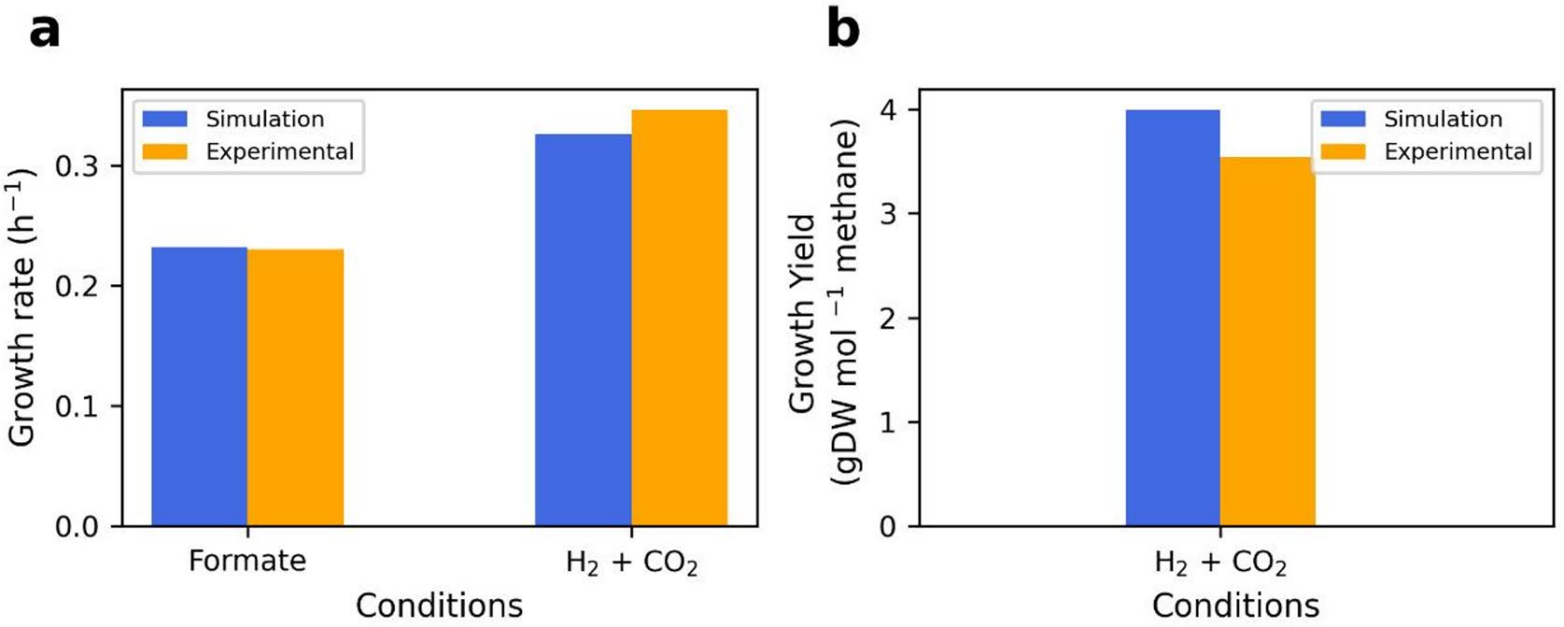
Comparison of the predicted and measured maximum growth rates under formate without H _2_ and CO_2_ with H_2_. The blue and orange colors refer to the model predictions and experimental data^12,47^, respectively. (**a**) Comparison of the predicted and measured growth rates (h^−1^) when the growth medium contained formate without H_2_ and CO_2_ with H_2_. (**b**) Comparison of the predicted and measured growth yield (gDW mol^−1^ methane) when the growth medium contained CO_2_ with H_2_.

To estimate the maximum growth rate under the formate batch condition, we used the UP cost ratio, GAM, and NGAM values used in chemostat simulations between the dilution rates of 0.002 and 0.1 per hour (Table 2). The predicted specific maximum growth rate was lower than the measured rate (Supplementary Fig. S15). As the model grew at higher growth rates, more nutrients were required and the saturation factors increased^8^. Therefore, the UP cost ratio was set to 44% of the total protein (Table 2). The model predicted a specific maximum growth rate of 0.23 per hour under the formate batch condition (Fig. 5a), consistent with the measured data^12^. As we had no measured yield at the maximum growth rate under the formate batch condition, the predicted yield was not in Fig. 5b.

To estimate the maximum growth rate under the CO₂ with H₂ batch condition, we decreased the GAM value to 25 mmol ATP gDW^− 1^. We also reduced the UP cost ratio from 44% to 20% of the total protein because the ratio of 44% was estimated at a growth rate of 0.23 per hour^12^. The model predicted a specific maximum growth rate and a growth yield of 0.32 per hour and 3.98 gDW mol^− 1^ methane, respectively (Fig. 5).

### The pcMMP model predicts the fitness of mutants

Knockout studies on *M. maripaludis* have addressed the role of H₂ in methanogenesis. Lupa et al.^48^ reported that *M. maripaludis* produced H₂ when the growth medium contained formate as the sole carbon source and electron donor. Lie et al.^14^ expected that *M. maripaludis* could grow without producing H₂. To this end, they genetically deleted the formate-hydrogen lyase. First, they generated two mutants (Δ3H₂ase and Δ5H₂ase) and measured their growth curves. Under formate-dependent conditions, they found that the Δ3H₂ase mutant could grow, while the Δ5H₂ase mutant could not. However, when they added H_2_ to the growth medium, the Δ5H₂ase mutant could grow. Finally, they generated a new Δ6H₂ase mutant from the Δ5H₂ase mutant by genetically deleting the *ehbN* gene to reveal the essential role of H_2_ in the growth of the *∆*6H_2_ase mutant.

**Fig. 6:**
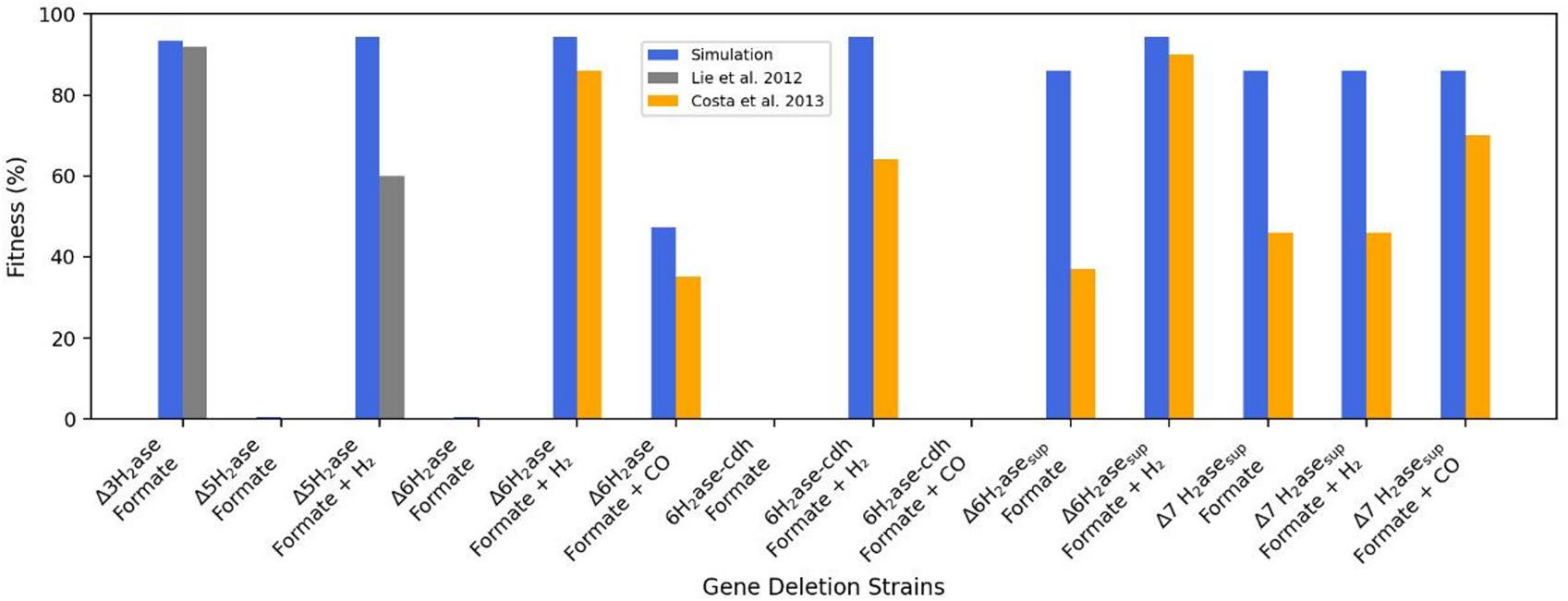
Comparison of the predicted and measured fitness of the different mutants. Blue refers to predicted fitness. The gray and orange colors refer to the experimental fitness from knockout studies: Lie et al.^14^ and Costa et al.^13^.

We simulated the Δ3H₂ase and Δ5H₂ase mutants using the pcMMP model (Fig. 6). Under formate-dependent conditions, this model predicted the fitness of the Δ3H₂ase mutant to be 93%, whereas the measured fitness was approximately 92%. The model also predicted no growth of the Δ5H₂ase mutant because of the small turnover rate of CODH used in the model (0.002 per second)^49^. However, the iMR539 model predicted that the Δ5H₂ase mutant grew under these conditions. Finally, we allowed the model to take up H₂. This increased the predicted fitness to 94%, whereas the measured fitness was approximately 60% (Supplementary Table S5).

Costa et al.^13^ studied two alternative pathways to demonstrate the non-requirement of H_2_ for the growth of *M. maripaludis*. They reported that the Δ6H₂ase mutant could grow with formate and CO in the absence of H_2_. This result indicates that *M. maripaludis* can utilize the reverse reaction of ACS/CODH, using CO oxidation to CO₂. To validate this pathway, they generated a new ∆6H_2_ase-∆*cdh* mutant by deleting the *cdh* gene and found that it could not grow under formate alone or with CO conditions. In the second pathway, they generated a new Δ6H₂ases_sup_ mutant by searching for mutations that can activate GAPOR. This mutant grew with formate as the sole electron donor and contained another hydrogenase (Eha). Therefore, they generated a new mutant (Δ7H₂ase_sup_) by genetically eliminating the *ehaN* and *ehaO* subunits in the Eha hydrogenase complex. They found that the Δ7H₂ase_sup_ mutant could grow with formate as the sole carbon source and electron donor.

The pcMMP model predicted no growth of the Δ6H₂ase mutant under formate-dependent conditions (Fig. 6). We then simulated the model with two different conditions. First, when we allowed the model to take up H_2_, the predicted fitness increased to 94%, whereas the measured fitness was 86%. Second, when we allowed the model to take up CO, the predicted fitness was 47%, whereas the measured fitness was 35%. For the ∆6H_2_ase-∆*cdh* mutant, the model predicted no growth under formate alone or with CO conditions. However, when we allowed the model to take up formate and H₂, the predicted fitness was 94%, whereas the measured fitness was 64%.

For the Δ6H₂ase_sup_ mutant growing under formate-dependent conditions, the pcMMP model predicted a fitness of 86%, whereas the measured fitness was 37%. When the model was allowed to take up H₂, the predicted fitness increased to 94%, whereas the measured fitness was 90%. The pcMMP model also predicted a constant fitness (86%) of the Δ7H₂ase_sup_ mutant under formate alone, formate with H_2_, or formate with CO conditions. However, the measured fitness of the Δ7H₂ase_sup_ mutant did not change under formate alone or with H_2_ conditions, whereas it increased slightly under formate with CO conditions (Fig. 6).

These results demonstrate the ability of the pcMMP model to predict the fitness of different mutants, whereas the iMR539 model can only predict growth or no growth (Supplementary Table S5).

### pcMMP limitations

Under formate-limited simulations, we fixed the value of $\:AA\:per\:ribosome$ to predict a constant ribosomal proteome fraction at different growth rates. However, under phosphate-limited simulations, we decreased the value of $\:AA\:per\:ribosome$ with the growth rate to predict the increase in ribosomal proteome fractions. The model predictions indicate that the value of $\:AA\:per\:ribosome$ plays a major role in determining the relationship between the ribosomal proteome fraction and growth rate. To explain this role, we can rewrite Eq. (2) as follows:

5 $$P=\frac{{m}_{aa}\:.\:R\:{.\:f}_{rRNA}\:}{{m}_{rr}}\:AA\:per\:ribosome$$

Equation (5) represents a nonlinear function of three variables: protein, RNA, and $\:AA\:per\:ribosome$. Mueller et al.^40^ reported that the $\:R/P$ ratio is a nonlinear function of growth rate using measured data from different organisms. Equation (5) contains two important unmodeled constraints. The first constraint controls the value of $\:AA\:per\:ribosome.$ In our simulations, we avoided this constraint by fixing $\:AA\:per\:ribosome$ values. The second constraint determines the protein mass under specific growth conditions. For example, at a growth rate of 0.003 per hour, *M. maripaludis* can produce 0.58 and 0.37 g protein per gDW under formate- and phosphate-limited conditions, respectively. We also avoided this constraint by changing the UP cost ratio. Therefore, the reconstruction of pc-models requires proteomics and biomass composition data sets at different growth rates to estimate these two important parameters^8,9^.

Mueller et al.^40^ reported that the organism *Thermoanaerobacter kivui* has different proteome allocation strategies than *E. coli* and *M. maripaludis*. *T. kivui* produced more ribosomal proteins that were not assembled with rRNA at low growth rates. Mueller et al.^40^ used the $\:R/P$ ratio as an indicator of ribosome abundance. They also compared this ratio in these three organisms. They reported that *M. maripaludis* exhibited a constant $\:R/P$ ratio at low growth rates, which was consistent with the fixed value of $\:AA\:per\:ribosome$ in our simulations. However, *T. kivui* exhibited a higher rate of change in the *R/P* ratio than *E. coli*. Therefore, the generation of new growth-dependent proteomics data sets for different organisms can provide new opportunities for proteome-constrained models to formulate new constraints that can determine protein mass and $\:AA\:per\:ribosome$ parameters.

The model predicted no growth under specific conditions. However, its predicted fitness was higher than the measured fitness in 7 of the 14 simulations. The higher predicted fitness indicates that these mutants may grow faster using adaptive laboratory evolution (ALE)^50^. For example, ALE has been used to increase the specific maximum growth rate of budding yeast grown on galactose and glycerol^51,52^. Under glycerol conditions, the specific maximum growth rate increased from 0.057 to 0.22 per hour^51^, whereas under galactose conditions, the specific maximum growth rate increased from 0.21 to 0.26 per hour^52^.

Costa et al.^13^ overexpressed GAPOR in mutants (Δ6H₂ase_sup_ and Δ7H₂ase_sup_), by finding mutations in the promoter upstream of the GAPOR gene. Consequently, the GAPOR abundances in their experiments may not match the predicted growth rates. We expected that the pcMMP model would produce a higher GAPOR abundance than that observed experimentally. Therefore, we simulated the model by limiting GAPOR abundances to 1% of the total protein. The predicted fitness was consistent with the measured fitness under this constraint (Supplementary Fig. S16). Therefore, we expected that ALE could increase the fitness of these mutants.

## Conclusion

The pcMMP model, a proteome-constrained model of the archaeon *M. maripaludis*, can be used as a knowledge base for archaeal systems biology. pcMMP integrated proteome constraints with the iMR539 metabolic model to study the special proteome allocation in *M. maripaludis* and the proteome constraints on maximum growth. The UP cost ratio and ribosomal turnover rate parameters were estimated from previously published experimental proteomic data. Therefore, the model can predict the biomass compositions at different growth rates, and the proteome constraints allowed prediction of the effect of enzymatic turnover rates on the growth rates of knockout mutants. Interestingly, pcMMP can compute the protein cost and predict a static ribosomal proteome fraction across growth rates, which differs between yeast and bacteria. Therefore, the proposed model can be used to study archaeal physiology, explore energy conservation, and provide a platform for optimizing methane production, metabolic engineering, and synthetic biology applications in archaea.

## Supplementary Information

Below is the link to the electronic supplementary material.

Supplementary Material 1

Supplementary Material 2

## Data Availability

The pcMMP model, SBML file, and simulation scripts are available on GitHub ([https://github.com/GhadaSamir2/pcMMP-model](https:/github.com/GhadaSamir2/pcMMP-model)). All collected data are included in the supplementary materials of this study.

## Abbreviations

GAM: Growth-associated maintenance
GEM: Genome-scale metabolic model
NGAM: Non-growth-associated maintenance
ORF: Open reading frame
pc: Proteome-constrainted
UP: Unspecific protein
