# Supplementary material for: Whole-cell modeling predicts alternative proteome allocation strategies in the archaeon Methanococcus maripaludis
Source: Sci Rep. 2026 Feb 19;16:7386. doi: 10.1038/s41598-026-37887-z (PMC12923902; doi:10.1038/s41598-026-37887-z)
Supplement: Supplementary file 1 — Supplementary Material 1 [file 41598_2026_37887_MOESM1_ESM.pdf]

Supplementary Information

Part 1: Steps required to convert iMR539 to pcMMP model

Step 1: Splitting isozymes and reversible reaction

Each reaction that had a "or" in the Gene-Protein-Reaction (GPR) association was split into multiple reactions, each of which was catalyzed by a distinct isozyme. In addition, we divided the reversible reactions into forward and reverse reactions. For example, the hydrogen acceptor oxidoreductase reaction is reversible and catalyzed by two isozyme complexes (mmp1382 and mmp1383 and mmp1384 and mmp1385) or (mmp0817 and mmp0818 and mmp0819 and mmp0820).

Reaction ID: rxn06299[c0]
Reaction Formula: H2[c0] + Coenzyme_F420[c0] <=> Reduced_coenzyme_F420[c0]
Catalyst: (mmp1382 and mmp1383 and mmp1384 and mmp1385) or (mmp0817 and mmp0818 and mmp0819 and mmp0820)

We spilt the reaction into four forward and reverse reactions, each catalyzed by a distinct isozyme complex.

Reaction ID: R360
Reaction Formula: Coenzyme_F420[c0] + H2[c0] --> Reduced_coenzyme_F420[c0]
Catalyst: mmp1382 and mmp1383 and mmp1384 and mmp1385

Reaction ID: R361
Reaction Formula: Reduced_coenzyme_F420[c0] --> Coenzyme_F420[c0] + H2[c0]
Catalyst: mmp1382 and mmp1383 and mmp1384 and mmp1385

Reaction ID: R362
Reaction Formula: Coenzyme_F420[c0] + H2[c0] --> Reduced_coenzyme_F420[c0]
Catalyst: mmp0817 and mmp0818 and mmp0819 and mmp0820

Reaction ID: R363
Reaction Formula: Reduced_coenzyme_F420[c0] --> Coenzyme_F420[c0] + H2[c0]
Catalyst: mmp0817 and mmp0818 and mmp0819 and mmp0820

Step 2: Complex formation process

For each complex, we constructed a formation reaction based on subunit stoichiometry compiled from structural data available in the PDB database (Supplementary Data 2). For example, a hydrogen acceptor oxidoreductase complex comprises mmp0817, mmp0818, mmp0819, and mmp0820.

Reaction ID: R2379

Reaction Formula: 12.0 MMP0817_peptide + 12.0 MMP0818_peptide + MMP0819_peptide + 12.0 MMP0820_peptide --> MMP0817_MMP0818_MMP0819_MMP0820_complex

Step 3: Complex dilution

Dilution reactions were added for each enzyme complex. This example represents the dilution reaction for the MMP0817_MMP0818_MMP0819_MMP0820_complex.

Reaction ID: R2380

Reaction Formula: MMP0817_MMP0818_MMP0819_MMP0820_complex -->

Step 4: The translation process

This process is divided into three steps: initiation, elongation, and termination. Initiation requires two GTP molecules[1], whereas elongation requires two GTP molecules for each amino acid added[2]. Termination requires one GTP[2]. For simplicity, we lumped the three steps into one translation reaction. Additionally, we ignored the selenocysteine (Sec, represented as "U") in the translation process. The following example represents the translation reaction of the MMP0819 gene.

Reaction ID: R1651

Reaction Formula: 333.0 GTP[c0] + 333.0 H2O[c0] + 4.0 ala-tRNA-GCA + ala-tRNA-GCC + 5.0 ala-tRNA-GCU + asn-tRNA-AAC + 7.0 asn-tRNA-AAU + 4.0 asp-tRNA-GAC + 13.0 asp-tRNA-GAU + 2.0 cys-tRNA-UGC + gln-tRNA-CAA + 2.0 gln-tRNA-CAG + 13.0 glu-tRNA-GAA + 8.0 gly-tRNA-GGA + 2.0 gly-tRNA-GGC + 2.0 gly-tRNA-GGG + gly-tRNA-GGU + 3.0 his-tRNA-CAC + his-tRNA-CAU + 7.0 ile-tRNA-AUA + 3.0 ile-tRNA-AUC + 9.0 ile-tRNA-AUU + imet-tRNA-AUG + 2.0 leu-tRNA-CUA + 2.0 leu-tRNA-CUC + 6.0 leu-tRNA-CUU + 9.0 leu-tRNA-UUA + 2.0 leu-tRNA-UUG + 13.0 lys-tRNA-AAA + 2.0 lys-tRNA-AAG + 2.0 met-tRNA-AUG + 3.0 phe-tRNA-UUU + 4.0 pro-tRNA-CCA + pro-tRNA-CCC + 3.0 pro-tRNA-CCU + 4.0 ser-tRNA-UCA + ser-tRNA-UCC + ser-tRNA-UCU + 2.0 thr-tRNA-ACG + thr-tRNA-ACU + tyr-tRNA-UAC + 5.0 tyr-tRNA-UAU + 5.0 val-tRNA-GUA + val-tRNA-GUC + val-tRNA-GUG + 5.0 val-tRNA-GUU --> 333.0 GDP[c0] + 333.0 H[c0] + MMP0819_peptide + 333.0 Phosphate[c0] + 13.0 tRNA-AAA + tRNA-AAC + 2.0 tRNA-AAG + 7.0 tRNA-AAU + 2.0 tRNA-ACG + tRNA-ACU + 7.0 tRNA-AUA + 3.0 tRNA-AUC + 2.0 tRNA-AUG + 9.0 tRNA-AUU + tRNA-CAA + 3.0 tRNA-CAC + 2.0 tRNA-CAG + tRNA-CAU + 4.0 tRNA-CCA + tRNA-CCC + 3.0 tRNA-CCU + 2.0 tRNA-CUA + 2.0 tRNA-CUC + 6.0 tRNA-CUU + 13.0 tRNA-GAA + 4.0 tRNA-GAC + 13.0 tRNA-GAU + 4.0 tRNA-GCA + tRNA-GCC + 5.0 tRNA-GCU + 8.0 tRNA-GGA + 2.0 tRNA-GGC + 2.0 tRNA-GGG + tRNA-GGU + 5.0 tRNA-GUA + tRNA-GUC + tRNA-GUG + 5.0 tRNA-GUU + tRNA-UAC + 5.0 tRNA-UAU + 4.0 tRNA-UCA + tRNA-UCC + tRNA-UCU + 2.0 tRNA-UGC + 9.0 tRNA-UUA + 2.0 tRNA-UUG + 3.0 tRNA-UUU + tRNAi-AUG

Step 5: the translation factors

In this step, we compiled translation factors from a previous study[3]. This example shows the translation reaction of the initiation factor MMP0603.

Reaction ID: R2880

Reaction Formula: 209.0 GTP[c0] + 209.0 H2O[c0] + ala-tRNA-GCA + 9.0 arg-tRNA-AGA + 2.0 arg-tRNA-AGG + 3.0 arg-tRNA-CGA + 3.0 asn-tRNA-AAU + 4.0 asp-tRNA-GAC + 3.0 asp-tRNA-GAU + cys-tRNA-UGC + cys-tRNA-UGU + 6.0 gln-tRNA-CAA + gln-tRNA-CAG + 6.0 glu-tRNA-GAA + glu-tRNA-GAG + 6.0 gly-tRNA-GGA + gly-tRNA-GGC + gly-tRNA-GGU + 3.0 ile-tRNA-AUC + 2.0 ile-tRNA-AUU + imet-tRNA-AUG + leu-tRNA-CUC + 2.0 leu-tRNA-CUU + 3.0 leu-tRNA-UUA + 5.0 lys-tRNA-AAA + 2.0 lys-tRNA-AAG + 4.0 met-tRNA-AUG + phe-tRNA-UUC + 2.0 pro-tRNA-CCA + pro-tRNA-CCC + pro-tRNA-CCG + 2.0 pro-tRNA-CCU + ser-tRNA-AGC + ser-tRNA-UCU + 3.0 thr-tRNA-ACA + thr-tRNA-ACC + thr-tRNA-ACU + 4.0 trp-tRNA-UGG + tyr-tRNA-UAC + tyr-tRNA-UAU + 5.0 val-tRNA-GUA + 7.0 val-tRNA-GUU --> 209.0 GDP[c0] + 209.0 H[c0] + MMP0603_peptide + 209.0 Phosphate[c0] + 5.0 tRNA-AAA + 2.0 tRNA-AAG + 3.0 tRNA-AAU + 3.0 tRNA-ACA + tRNA-ACC + tRNA-ACU + 9.0 tRNA-AGA + tRNA-AGC + 2.0 tRNA-AGG + 3.0 tRNA-AUC + 4.0 tRNA-AUG + 2.0 tRNA-AUU + 6.0 tRNA-CAA + tRNA-CAG + 2.0 tRNA-CCA + tRNA-CCC + tRNA-CCG + 2.0 tRNA-CCU + 3.0 tRNA-CGA + tRNA-CUC + 2.0 tRNA-CUU + 6.0 tRNA-GAA + 4.0 tRNA-GAC + tRNA-GAG + 3.0 tRNA-GAU + tRNA-GCA + 6.0 tRNA-GGA + tRNA-GGC + tRNA-GGU + 5.0 tRNA-GUA + 7.0 tRNA-GUU + tRNA-UAC + tRNA-UAU + tRNA-UCU + tRNA-UGC + 4.0 tRNA-UGG + tRNA-UGU + 3.0 tRNA-UUA + tRNA-UUC + tRNAi-AUG

Step 6: Unspecific Protein (UP)

To represent proteins not included in the pcMMP model, we added a reaction for UP. The amino acids composition of this reaction was the same as that in the biomass equation used in the iMR539 model[4].

Reaction ID: R2871

Reaction Formula: 921.0 GTP[c0] + 921.0 H2O[c0] + 9.0 ala-tRNA-GCA + 8.0 ala-tRNA-GCC + 18.0 ala-tRNA-GCG + 9.0 ala-tRNA-GCU + 3.0 arg-tRNA-AGA + 5.0 arg-tRNA-AGG + 3.0 arg-tRNA-CGA + 5.0 arg-tRNA-CGC + 5.0 arg-tRNA-CGG + 4.0 arg-tRNA-CGU + 13.0 asn-tRNA-AAC + 8.0 asn-tRNA-AAU + 10.0 asp-tRNA-GAC + 11.0 asp-tRNA-GAU + 4.0 cys-tRNA-UGC + 4.0 cys-tRNA-UGU + 11.0 gln-tRNA-CAA + 12.0 gln-tRNA-CAG + 13.0 glu-tRNA-GAA + 10.0 glu-tRNA-GAG + 11.0 gly-tRNA-GGA + 16.0 gly-tRNA-GGC + 14.0 gly-tRNA-GGG + 11.0 gly-tRNA-GGU + 3.0 his-tRNA-CAC + 5.0 his-tRNA-CAU + 10.0 ile-tRNA-AUA + 6.0 ile-tRNA-AUC + 9.0 ile-tRNA-AUU + imet-tRNA-AUG + 6.0 leu-tRNA-CUA + 6.0 leu-tRNA-CUC + 9.0 leu-tRNA-CUG + 4.0 leu-tRNA-CUU + 8.0 leu-tRNA-UUA + 5.0 leu-tRNA-UUG + 16.0 lys-tRNA-AAA + 13.0 lys-tRNA-AAG + 12.0 met-tRNA-AUG + 7.0 phe-tRNA-UUC + 9.0 phe-tRNA-UUU + 6.0 pro-tRNA-CCA + 5.0 pro-tRNA-CCC + 2.0 pro-tRNA-CCG + 6.0 pro-tRNA-CCU + 4.0 ser-tRNA-AGC + 2.0 ser-tRNA-AGU + 2.0 ser-tRNA-UCA + 3.0 ser-tRNA-UCC + 3.0 ser-tRNA-UCG + 5.0 ser-tRNA-UCU + 6.0 thr-tRNA-ACA + 7.0 thr-tRNA-ACC + 4.0 thr-tRNA-ACG + 5.0 thr-tRNA-ACU + 5.0 trp-tRNA-UGG + 5.0 tyr-tRNA-UAC + 8.0 tyr-tRNA-UAU + 8.0 val-tRNA-GUA + 14.0 val-tRNA-GUC + 7.0 val-tRNA-GUG + 7.0 val-tRNA-GUU --> 921.0 GDP[c0] + 921.0 H[c0] + 921.0 Phosphate[c0] + 16.0 tRNA-AAA + 13.0 tRNA-AAC + 13.0 tRNA-AAG + 8.0 tRNA-AAU + 6.0 tRNA-ACA + 7.0 tRNA-ACC + 4.0 tRNA-ACG + 5.0 tRNA-ACU + 3.0 tRNA-AGA + 4.0 tRNA-AGC + 5.0 tRNA-AGG + 2.0 tRNA-AGU + 10.0 tRNA-AUA + 6.0 tRNA-AUC + 12.0 tRNA-AUG + 9.0 tRNA-AUU + 11.0 tRNA-CAA + 3.0 tRNA-CAC + 12.0 tRNA-CAG + 5.0 tRNA-CAU + 6.0 tRNA-CCA + 5.0 tRNA-CCC + 2.0 tRNA-CCG + 6.0 tRNA-CCU + 3.0 tRNA-CGA + 5.0 tRNA-CGC + 5.0 tRNA-CGG + 4.0 tRNA-CGU + 6.0 tRNA-CUA + 6.0 tRNA-CUC + 9.0 tRNA-CUG + 4.0 tRNA-CUU + 13.0 tRNA-GAA + 10.0 tRNA-GAC + 10.0 tRNA-GAG + 11.0 tRNA-GAU + 9.0 tRNA-GCA + 8.0 tRNA-GCC + 18.0 tRNA-GCG + 9.0 tRNA-GCU + 11.0 tRNA-GGA + 16.0 tRNA-GGC + 14.0 tRNA-GGG + 11.0 tRNA-GGU + 8.0 tRNA-GUA + 14.0 tRNA-GUC + 7.0 tRNA-GUG + 7.0 tRNA-GUU + 5.0 tRNA-UAC + 8.0 tRNA-UAU + 2.0 tRNA-UCA + 3.0 tRNA-UCC + 3.0 tRNA-UCG + 5.0 tRNA-UCU + 4.0 tRNA-UGC + 5.0 tRNA-UGG + 4.0 tRNA-UGU + 8.0 tRNA-UUA + 7.0 tRNA-UUC + 5.0 tRNA-UUG + 9.0 tRNA-UUU + tRNAi-AUG + up_protein

Step 7: UP dilution

Reaction ID: R2872

Reaction Formula: up_protein -->

Step 8: Ribosome

Ribosomes are complex machines consisting of both ribosomal proteins and rRNA that were collected from the KEGG database. First, we added individual reactions for large and small ribosomal subunits and rRNA. Then, we compiled the ribosomal proteins and rRNA into a single reaction to represent the ribosome formation.

Reaction ID: R2061

Reaction Formula: 16S + 23S + 5S + MMP0060_peptide + MMP0062_peptide + MMP0093_peptide + MMP0151_peptide + MMP0156_peptide + MMP0159_peptide + MMP0249_peptide + MMP0258_peptide + MMP0259_peptide + MMP0260_peptide + MMP0298_peptide + MMP0443_peptide + MMP0444_peptide + MMP0574_peptide + MMP0577_peptide + MMP0625_peptide + MMP0627_peptide + MMP0639_peptide + MMP0640_peptide + MMP0641_peptide + MMP0667_peptide + MMP0669_peptide + MMP1147_peptide + MMP1207_peptide + MMP1289_peptide + MMP1319_peptide + MMP1320_peptide + MMP1321_peptide + MMP1323_peptide + MMP1324_peptide + MMP1325_peptide + MMP1365_peptide + MMP1367_peptide + MMP1368_peptide + MMP1371_peptide + MMP1403_peptide + MMP1404_peptide + MMP1405_peptide + MMP1408_peptide + MMP1409_peptide + MMP1410_peptide + MMP1411_peptide + MMP1412_peptide + MMP1413_peptide + MMP1414_peptide + MMP1415_peptide + MMP1416_peptide + MMP1417_peptide + MMP1418_peptide + MMP1419_peptide + MMP1420_peptide + MMP1421_peptide + MMP1433_peptide + MMP1543_peptide + MMP1544_peptide + MMP1545_peptide + MMP1546_peptide + MMP1547_peptide + MMP1579_peptide + MMP1658_peptide + MMP1708_peptide + MMP1709_peptide --> ribosome

Step 9: Ribosome dilution

Reaction ID: R2062

Reaction Formula: ribosome -->

Step 10: Transcription of mRNA

For simplicity, we represented transcription in our model as a single reaction without separating the steps, and the initiation step was not included. The reaction consumes ATP, CTP, GTP, and UTP to produce the mRNA for each gene, along with pyrophosphate (PPi) as a byproduct[2]. This example shows the transcription reaction of the MMP0819 gene.

Reaction ID: R3525

Reaction Formula: 186.0 ATP[c0] + 74.0 CTP[c0] + 94.0 GTP[c0] + 147.0 UTP[c0] --> MMP0819_mRNA + 501.0 PPi[c0]

Step 11: Degradation of mRNA

In the pcMMP model, we added an mRNA degradation reaction similar to the model for *Lactococcus lactis* (*L. lactis*)[2]. The first nucleotide is converted back into a nucleoside triphosphate. The remaining nucleotides in the reaction are hydrolyzed into nucleoside monophosphate. Additionally, one ATP was required for every four nucleotides. This example shows the mRNA degradation reaction of the MMP0819 gene.

Reaction ID: R3526

Reaction Formula: 125.0 ATP[c0] + 626.0 H2O[c0] + MMP0819_mRNA --> 126.0 ADP[c0] + 185.0 AMP[c0] + 74.0 CMP[c0] + 94.0 GMP[c0] + 626.0 H[c0] + 126.0 Phosphate[c0] + 147.0 UMP[c0]

Step 12: Dilution of mRNA

Reaction ID: R3527

Reaction Formula: MMP0819_mRNA -->

Step 13: Ribosomal RNA (rRNA)

rRNA, including 16S, 23S, and 5S, is transcribed and assembled with ribosomal protein to form ribosomes. For example, 5S rRNA can be synthesized from four different genes. In our model, we added four genes and then used them to build the 5S. An example of this process is shown below for the RNA_37 gene.

Reaction ID: R2047
Reaction Formula: 29.0 ATP[c0] + 30.0 CTP[c0] + 27.0 GTP[c0] + 29.0 UTP[c0] --> 115.0 PPi[c0] + RNA_37

Reaction ID: R2048
Reaction Formula: RNA_37 --> 5S

Step 14: Transcription of tRNA

Reaction ID: R1426
Reaction Formula: 42.0 ATP[c0] + 37.0 CTP[c0] + 42.0 GTP[c0] + 43.0 UTP[c0] --> 164.0 PPi[c0] + tRNA-UGG

Step 15: Charging of tRNA

The process of tRNA charging is a key step in translation, where tRNA is charged with their corresponding amino acid to prepare it for participation in protein synthesis. The tRNA charging reaction requires ATP, which is hydrolyzed to AMP and pyrophosphate, as described in the KEGG database (Reaction ID: R03664). This example shows the charging of the tRNA with the amino acid tryptophan.

Reaction ID: R1427
Reaction Formula: ATP[c0] + L-Tryptophan[c0] + tRNA-UGG --> AMP[c0] + PPi[c0] + trp-tRNA-UGG

Step 16: Dilution of tRNA

Reaction ID: R4792

Reaction Formula: tRNA-UGG -->

Step 17: Glycogen

Key reactions for glycogen biosynthesis are missing in the iMR539 model[4]. Therefore, we added three reactions to fill the gaps in the pathway[5].

Reaction ID: R2873

Reaction Formula: D-glucose-6-phosphate[c0] --> Glucose-1-phosphate[c0]

Catalyst: mmp1372

Reaction ID: R2875

Reaction Formula: UDP-glucose[c0] --> Glycogen[c0] + UDP[c0]

Catalyst: mmp1294

A third reaction was added to enable glycogen export.

Reaction ID: R2879

Reaction Formula: Glycogen[c0] -->

Catalyst: _

Step 18: Biomass, GAM, and NGAM

We used the biomass reaction from the iMR539 model[4] and adjusted it by removing RNA, protein, and the Growth-Associated Maintenance (GAM) cost, which was then added as a new reaction and deactivated the original biomass reaction. Additionally, we added reactions for GAM and Non-Growth-Associated Maintenance (NGAM).

Reaction ID: R4799

Reaction Formula: 0.0030965 ACP[c0] + 0.0030965 Archaellin[e0] + 0.0030965 Ca2[c0] + 0.0030965 Calomide[c0] + 0.0030965 Cl-[c0] + 0.0030965 Co2[c0] + 0.0030965 CoA[c0] + 0.0030965 CoM[c0] + 0.0030965 Coenzyme_F420-3[c0] + 0.0030965 Coenzyme_F420[c0] + 0.0030965 FAD[c0] + 0.0030965 Factor_430[c0] + 0.0030965 Fe2[c0] + 0.0030965 GSH[c0] + 0.0030965 H4MPT[c0] + 0.0030965 HTP[c0] + 0.0030965 K[c0] + 0.0030965 Methanofuran[c0] + 0.0030965 Mg[c0] + 0.0030965 Molybdenum[c0] + 0.0030965 NADP[c0] + 0.0030965 NAD[c0] + 0.0030965 Putrescine[c0] + 0.0030965 Pyridoxal_phosphate[c0] + 0.0030965 Riboflavin[c0] + 0.0030965 S-Adenosyl-L-methionine[c0] + 0.0030965 Saturated_Archaetidylserine[c0] + 0.0030965 Saturated_CDP-archaeol[c0] + 0.0030965 Selenoprotein[c0] + 0.0030965 Siroheme[c0] + 0.0030965 Spermidine[c0] + 0.0030965 TPP[c0] + 0.021439 TTP[c0] + 0.021439 dATP[c0] + 0.010608 dCTP[c0] + 0.010608 dGTP[c0] + 0.0030965 fe3[c0] --> Biomass[c0] + 0.0030965 Cobinamide[c0] + 0.0030965 Dimethylbenzimidazole[c0] + 0.0030965 apo-ACP[c0]

Reaction ID: R4800

Reaction Formula: {GAM} ATP[c0] + {GAM} H2O[c0] --> {GAM} ADP[c0] + {GAM} H[c0] + {GAM} Phosphate[c0]

Reaction ID: R4801

Reaction Formula: ATP[c0] + H2O[c0] --> ADP[c0] + H[c0] + Phosphate[c0]

The free amino acid molecule

We simulated the free amino acid molecule by adding two new reactions: biosynthesis and dilution of the free amino acid molecule.

Reaction ID: R4802

Reaction Formula: 0.0286566875021862 L-Methionine[c0] + 0.0553177214928744 L-Arginine[c0] + 0.0959550569859981 L-Alanine[c0] + 0.0473283805249331 L-Threonine[c0] + 0.0450322578330521 L-Aspartate[c0] + 0.114328523135678 Glycine[c0] + 0.0640044200361819 L-Lysine[c0] + 0.0402404470981404 L-Serine[c0] + 0.0491267109925977 L-Glutamate[c0] + 0.0491267109925977 L-Glutamine[c0] + 0.0170742732906219 L-Cysteine[c0] + 0.0841739743462104 L-Leucine[c0] + 0.0270601646929878 L-Tyrosine[c0] + 0.0450322578330521 L-Asparagine[c0] + 0.0105841389943521 L-Tryptophan[c0] + 0.0413369353758062 L-Proline[c0] + 0.0542189909078923 L-Isoleucine[c0] + 0.0346481326513131 L-Phenylalanine[c0] + 0.0177734247118411 L-Histidine[c0] + 0.0789807906016828 L-Valine[c0] --> AA

Reaction ID: R4803

Reaction Formula: AA -->

Part 2: Model Description

The mass balance constraint

We used the main constraint in the flux balance analysis ($S.v = 0$), where S is the stochiometric matrix, and v is the vector of all fluxes in the model.

Enzyme capacity constraint

Assume that a metabolic reaction has a flux value v, and an enzyme complex E can catalyze this reaction. The pc-model[6] has a reaction v_e that assembles the complex E from its subunit peptides. Therefore, we can couple between the fluxes v and the abundances of enzyme [E] as:

$$v = k_{cat} . [E]. \sigma$$

Where the k_{cat} is the turnover rate for enzyme E and σ is the saturation factor, which is assumed to have the value one. Therefore, the pc-model estimated the minimum enzyme abundances that are required to match the metabolic flux. Additionally, at the steady state conditions, we can estimate [E] as:

$$\frac{dE}{dt} = v_e - \mu . [E] = 0$$

$$[E] = \frac{v_e}{\mu}$$

Therefore, the final enzyme capacity constraint can be rewritten as:

$$v = k_{cat} \cdot \frac{v_e}{\mu}$$

Ribosome capacity constraint

The pc-model contains a flux $v_{trans,i}$ that describes how a peptide i can be translated from charged tRNAs. The model must allocate a fraction of ribosomes eR_ithat can translate all abundances of peptide i. By using the ribosome turnover rate $k_{cat,ribo}$(aa/h) and the enzyme capacity constraint, we can get:

$$v_{trans,i} = \frac{k_{cat,ribo}}{\text{length}(\text{peptide}_i)} \cdot eR_i$$

Additionally, the model has the flux $v_{syn,ribo}$ that describes how ribosome is assembled from ribosomal protein and rRNA. Therefore, we can estimate ribosome abundances at the steady state as follows:

$$\frac{d\ Ribosome}{dt} = v_{syn,ribo} - \mu . [Ribosome] = 0$$

$$[Ribosome] = \frac{v_{syn,ribo}}{\mu}$$

Therefore, we write ribosome capacity constraint that enforces all fractions of ribosomes eR_i equals to the ribosome abundance in the model as follows:

$$\sum eR_i - \frac{1}{\mu} \cdot v_{syn,ribo} = 0$$

Additionally, we can use the flux $v_{syn,ribo}$ to estimate the rRNA mass as follows:

$$\frac{v_{syn,ribo}}{\mu} . \frac{mw_{rRNA}}{1000} - rRNA = 0$$

UP cost constraint

The pc-model contains UP that represents the protein abundances that are not in the model. The sequence of this protein represents the amino acid compositions in the biomass reaction in the metabolic model. The flux v_{UP} is the translational flux for peptide UP.

$$\frac{dUP}{dt} = v_e - \mu . [UP] = 0$$

$$[UP] = \frac{v_{UP}}{\mu}$$

Therefore, we can constrain the model to produce a specific UP mass (c) as follows:

$$\frac{v_{UP}}{\mu} . \frac{mw_{UP}}{1000} \geq c$$

Total proteome constraint

The unit of fluxes v_e, v_{ribo}, and v_{UP} is mmol/gDW/h, so we can estimate the mass of metabolic enzymes, ribosomes, and UP by multiplying with $\frac{mw}{1000}$, where mw is the molecular weight as follows:

$$\sum \frac{v_e}{\mu} . \frac{mw_e}{1000} + \left(\frac{v_{syn,ribo}}{\mu} . \frac{mw_{r-proteins}}{1000}\right) + \left(\frac{v_{UP}}{\mu} . \frac{mw_{UP}}{1000}\right) - Total\ Protein = 0$$

mRNA coupling constraint

The pc-model contains a flux $v_{transcription,i}$ that describes how an $mRNA_i$ can be transcribed from RNA nucleotides. The pc-model has a degradation reaction for $mRNA_i$ with degradation rate (k_{deg}). The abundance of $mRNA_i$ can be estimated at the steady state as follows:

$$\frac{d\,mRNA_i}{dt} = v_{transcription,i} - \mu . [mRNA_i] - k_{deg} . [mRNA_i] = 0$$

$$[mRNA_i] = \frac{v_{transcription,i}}{\mu + k_{deg}}$$

Additionally, we can use the flux $v_{transcription,i}$ to estimate the total mRNA mass using the molecular weight of each transcript of $mRNA_i$ as follows:

$$\sum \frac{v_{transcription,i}}{\mu + k_{deg}} . \frac{mw_{mRNA_i}}{1000} - mRNA = 0$$

Finally, the used the mRNA coupling constraint to couple between the translational flux $v_{trans,i}$ and $v_{transcription,i}$, as follows:

$$v_{trans,i} - \frac{k_{mRNA}}{k_{deg} + \mu} \cdot v_{transcription,i} = 0$$

Where k_{mRNA} is catalytic rate of mRNA (protein/mRNA/s)[2], estimated as follows:

$$k_{mRNA} = \frac{\mu\, P/m_{aa}}{R\, f_{mRNA}/m_{nt}}$$

Where f_{mRNA} is fraction of mRNA in total RNA, and m_{nt} is the molecular weight of the average nucleotide.

tRNA coupling constraint

The pc-model contains a flux $v_{tRNA\ charging,i}$ that describes how $tRNA_i$ is charged with an amino acid. Additionally, the model has a flux $v_{transcription,tRNA,i}$ that describes how a $tRNA_i$ can be transcribed from RNA nucleotides. The abundance of $tRNA_i$ can be estimated at the steady state as follows:

$$\frac{d\ tRNA_i}{dt} = v_{transcription,tRNA,i} - \mu . [tRNA_i] = 0$$

$$[tRNA_i] = \frac{v_{transcription,tRNA,i}}{\mu}$$

Additionally, we can use the flux $v_{transcription,tRNA,i}$ to estimate the total tRNA mass using the molecular weight of each transcript $tRNA_i$ as follows:

$$\sum \frac{v_{transcription,tRNA,i}}{\mu} . \frac{mw_{tRNA_i}}{1000} - tRNA = 0$$

Finally, the used the tRNA coupling constraint to couple between $v_{tRNA\ charging,i}$ and $v_{transcription,tRNA,i}$ as follows:

$$v_{tRNA\,charging,i} - \frac{k_{tRNA}}{\mu} \cdot v_{transcription,tRNA,i} = 0$$

Where k_{tRNA} is catalytic rate of tRNA (aa/tRNA/s)[2], estimated from the following equation:

$$k_{tRNA} = \frac{\mu\, P/m_{aa}}{R\, f_{tRNA}/m_{tRNA}}$$

Where f_{tRNA} is fraction of tRNA in total RNA, and m_{tRNA} is the molecular weight of the average tRNA.

Objective function

The model contains nonlinear functions with the growth rate μ. To solve the model as a linear programming solver, we first need to fix a value of μ. In the chemostat simulation, we minimized the formate uptake rate under the formate-limited conditions. In phosphate-limited conditions, we minimized the uptake rate of phosphate. In the batch simulations, we searched for the maximum growth of μ where the model has a feasible solution. Therefore, the uptake has no role in determining the maximum growth rate. We adopted this function from GECKO model[7].

Parameters

Parameter	Value	Reference
m_{aa} (average molecular weight of an amino acid)	109 g/mol	[2]
m_{nt} (average molecular weight of mRNA nucleotide)	324 g/mol	[2]
m_{rr} (rRNA mass per ribosome)	1444610 g/ mol_ribosome	this study
m_{tRNA} (average molecular weight of tRNA)	25000 g/mol	[2]
f_{rRNA} (rRNA fraction in RNA)	0.7961	[8]
f_{mRNA} (mRNA fraction in RNA)	0.1039	
f_{tRNA} (tRNA fraction in RNA)	0.10	[9]
P (total protein mass)	0.6 g/gDW	[8]
R (total RNA mass)	0.14 g/gDW	[8]
Catalytic rate of tRNA (aa/tRNA/s) $k_{tRNA} = \frac{\mu P/m_{aa}}{R f_{tRNA}/m_{tRNA}}$	2.73 μ	[2]
Catalytic rate of mRNA (protein/mRNA/s) $k_{mRNA} = \frac{\mu P/m_{aa}}{R f_{mRNA}/m_{nt}}$	0.034 μ	[2]
mRNA degradation constant k_{deg}	8.3 h^{-1}	[10]

Normally, the rRNA is about 0.85, tRNA is 0.10[9], and mRNA is 0.05 of the total RNA. As measured data, rRNA was 0.7961[8]. To maintain the total RNA fraction, the mRNA value was adjusted to 0.1039.

Template of the reactions

**Process**	**Reaction template**
Complex formation	$s_1\, peptide_1 + s_2\, peptide_2 + \ldots + s_k\, peptide_k \rightarrow peptide1\_peptide2\_\ldots\_peptideK\_complex$
Translation	$(2m+1)GTP + (2m+1)H_2O + \sum aa - tRNA - codon \rightarrow (2m+1)GDP + (2m+1)Pi + (2m+1)\, H^+ + \sum tRNA - codon + gene\_peptide$
Ribosome	$\sum rRNA + \sum RibosomalProtein \rightarrow Ribosome$
Transcription	$\sum_{i=1}^{n} NTP \rightarrow gene\_mRNA + n\, ppi$
Degradation of mRNA	$\left\lceil \frac{n}{4} \right\rceil ATP + (\left\lceil \frac{n}{4} \right\rceil + (n-1))\, H2O + gene\_mRNA \rightarrow \left\lceil \frac{n}{4} \right\rceil ADP + \sum_{i=1}^{n-1} NMP + 1\, NTP\_first + (\left\lceil \frac{n}{4} \right\rceil + (n-1))\, H^+ + \left\lceil \frac{n}{4} \right\rceil Pi$
Charging of tRNA	$ATP + AminoAcid + tRNA - codon \rightarrow AMP + PPi + aa - tRNA - codon$
Dilution	$Macromolecule \rightarrow \emptyset$

s_k: Stoichiometric of peptide subunit k in a protein complex

m: Number of amino acids in the protein

n: Number of nucleotides in the mRNA

Supplementary Figures

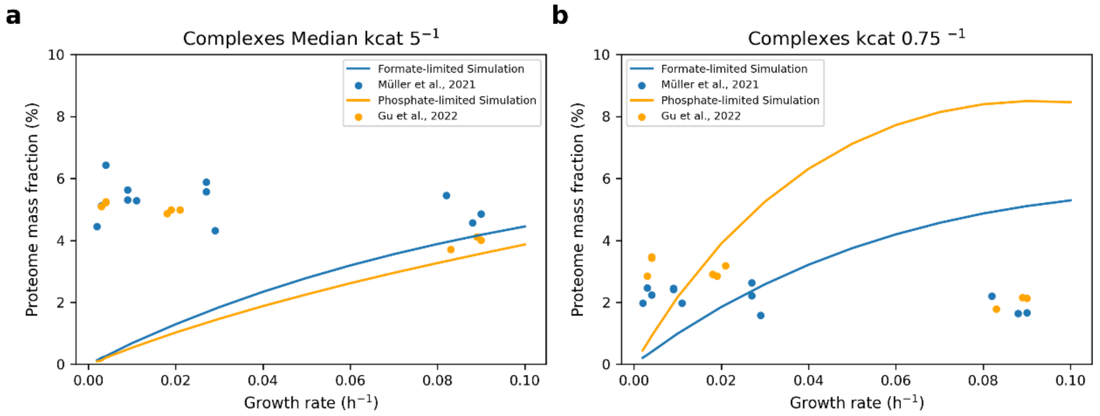

Figure S1. The predicted enzymatic complexes with fixed turnover rates in the model. **a** Comparison of the minimum abundances of all enzymatic complexes with a median turnover rate 5^{-1}. **b** Comparison of the minimum abundances of all enzymatic complexes with a fixed turnover rate 0.75 s^{-1}.

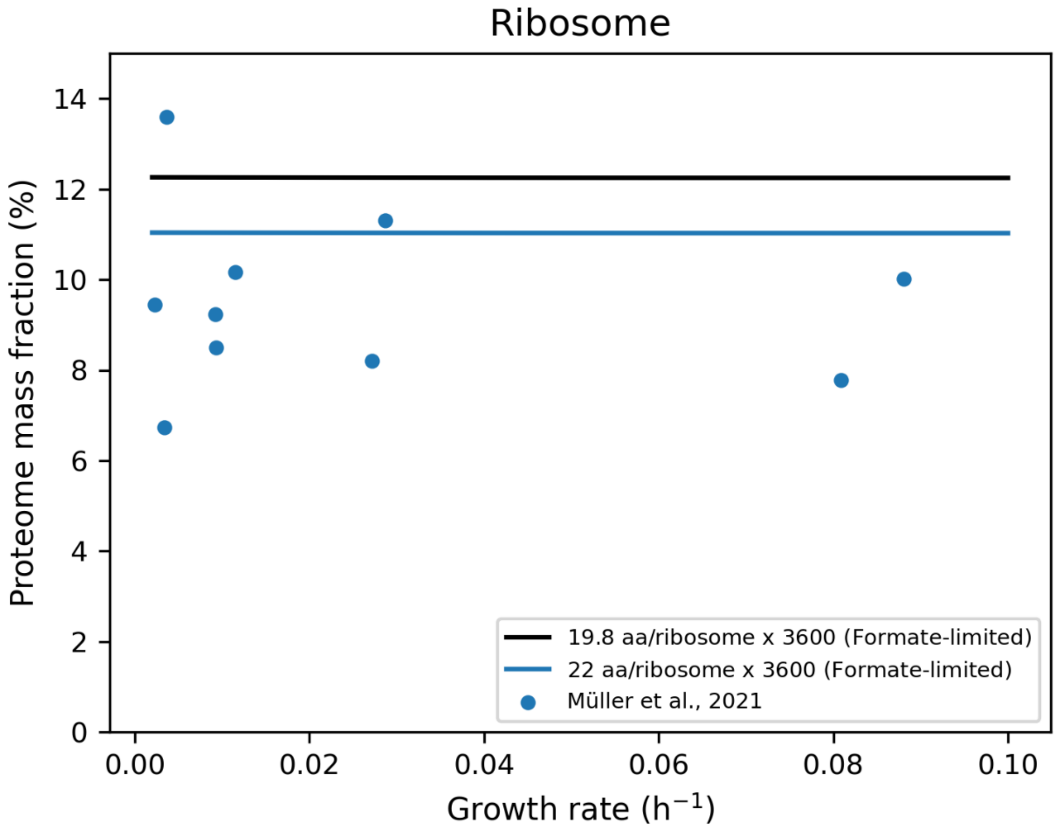

Figure S2. Effect of the parameter $AA\ per\ ribosome$ on ribosome abundance in the model under formate-limited conditions. Blue and black colors refer to the values of $AA\ per\ ribosome$: 22x3600 and 19.8x3600 (aa/ribosome), respectively. In our simulation, we used $AA\ per\ ribosome$ as 22x3600 (aa/ribosome) in simulations, except under phosphate-limited conditions.

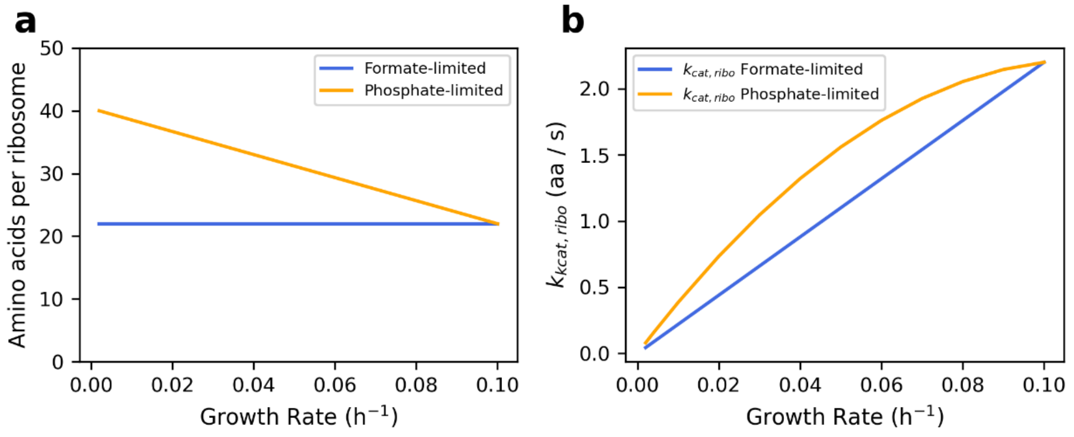

Figure S3. Estimation $AA\ per\ ribosome$, and $k_{cat,ribo}$ under formate- and phosphate-limited conditions. **a** Comparison $AA\ per\ ribosome$ under phosphate- and formate-limited conditions. The orange line refers to the regression line used in the simulation under phosphate-limited conditions. The blue line refers to the $AA\ per\ ribosome$ under formate-limited conditions. **b** Plotting curves for $k_{cat,ribo} = \mu\ .\ AA\ per\ ribosome$, under phosphate- and formate-limited conditions.

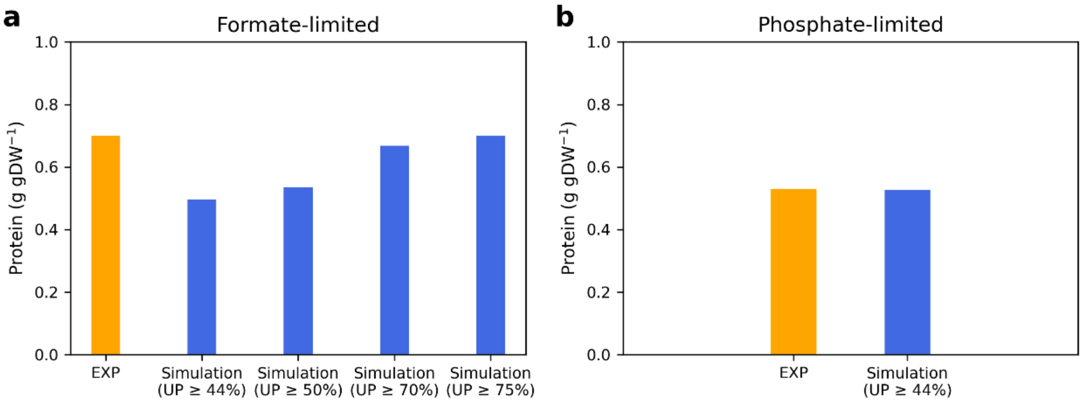

Figure S4. Estimation of the UP cost ratio under formate- and phosphate-limited conditions. The orange and blue lines refer to the measured data and model predictions, respectively. The UP cost ratio equals 44% was estimated at maximum growth rate 0.23 per hour under formate-limited conditions. **a** Effect of the UP cost ratio on the predicted total protein mass under formate-limited conditions. **b** Effect of the UP cost ratio on the predicted total protein mass under phosphate-limited conditions.

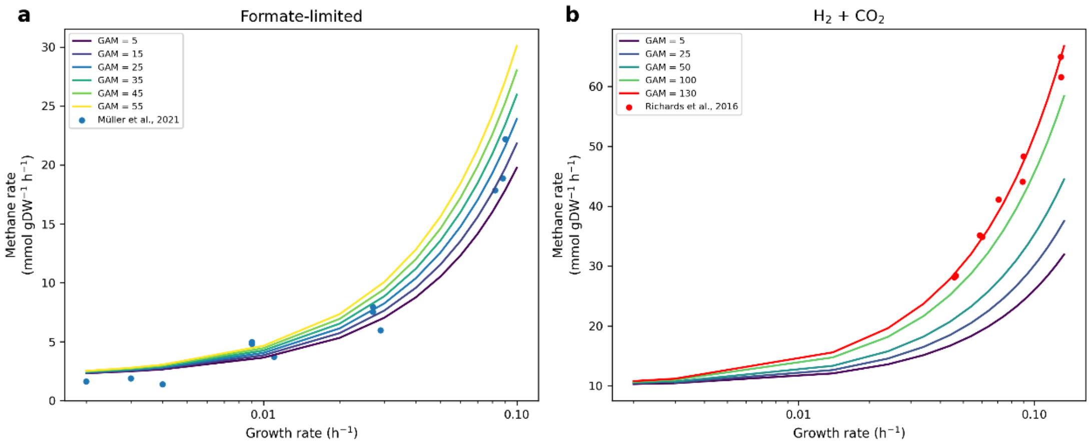

Figure S5. Effect of GAM values on methane fluxes. Methane fluxes increase with GAM. **a** Model predictions under formate-limited conditions with different GAM values. **b** Model predictions under CO_2-limited conditions with H_2 and different GAM values.

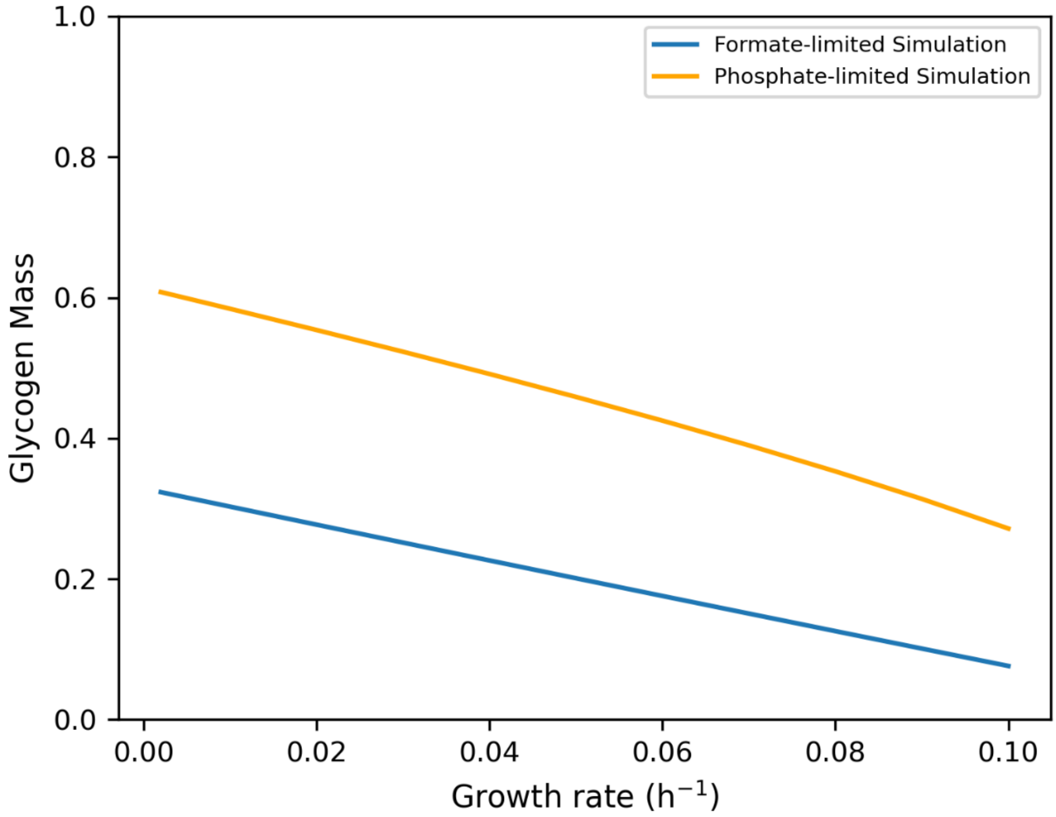

Figure S6. Glycogen mass under formate- and phosphate-limited conditions. Glycogen mass means the ability of the model to store carbon storage. The orange and blue lines are phosphate- and formate-limited conditions, respectively.

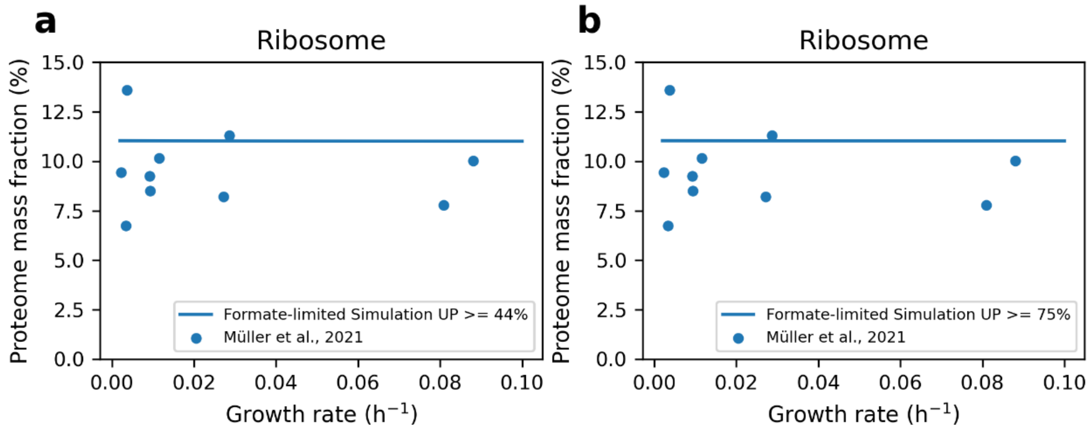

Figure S7. Effect of the UP cost ratio on ribosome abundance in the model. We simulated the model with values of UP cost ratio as 44% and 75% of the total protein mass. These results indicate that the UP cost ratio has no effect on ribosome abundance in the model. **a** Model simulation with UP $\geq$ 44%. **b** Model simulation with UP $\geq$ 75%.

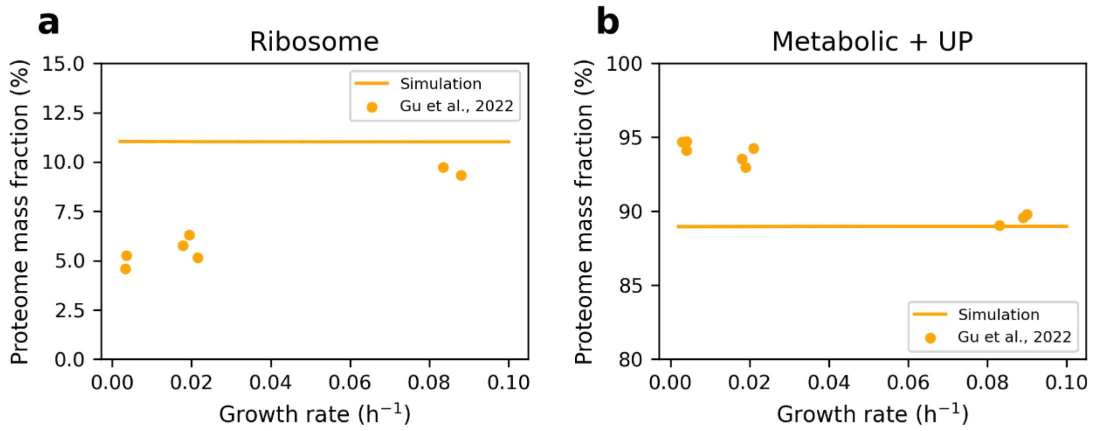

Figure S8. Effect of the *AA per ribosome* (fixed at 22x3600 amino acids per ribosome) on the proteome fractions in the model under phosphate-limited conditions. **a** Comparison between the predicted ribosomal proteome fractions and the measured data. **b** Comparison between the predicted sum of metabolic and UP proteome fractions and the measured data.

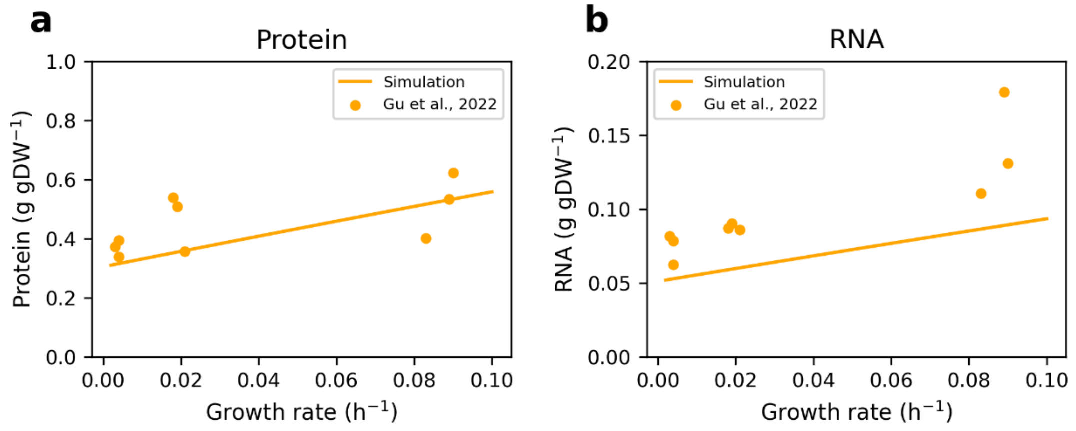

Figure S9. Effect of the *AA per ribosome* (fixed at 22x3600 amino acids per ribosome) on the predicted masses of protein and RNA under phosphate-limited conditions. **a** Comparison between the predicted protein mass and the measured data. **b** Comparison between the predicted RNA mass and the measured data.

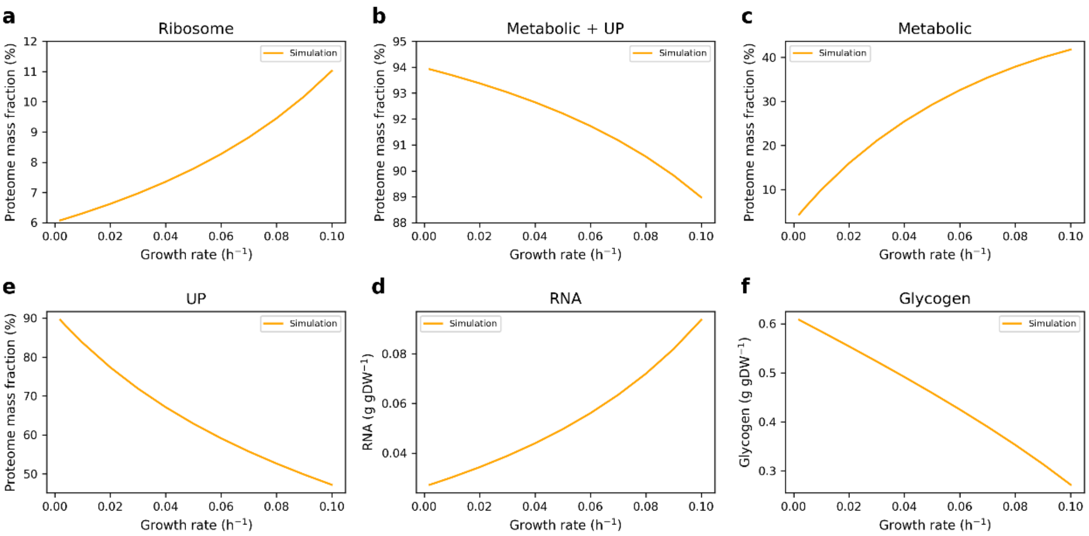

Figure S10. Change in the predicted biomass compositions under phosphate-limited conditions when the value of $AA\ per\ ribosome$ is changed (based on the regression line in Figure S3a) at growth rate.

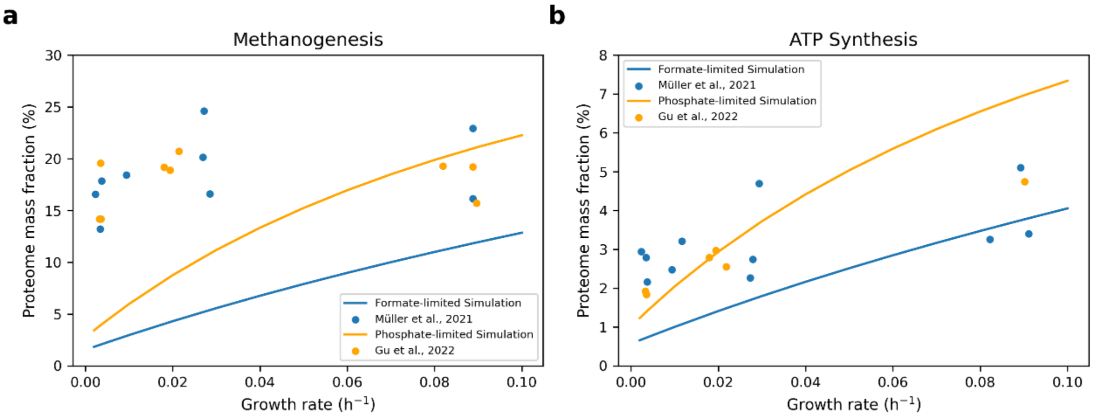

Figure S11. The predicted enzymatic complexes in the model. **a** Comparison of the summation of the predicted minimal enzyme abundances in the methanogenesis pathway with the measured proteome fractions. **b** Comparison of minimum ATP synthesis abundances with the measured proteome fractions.

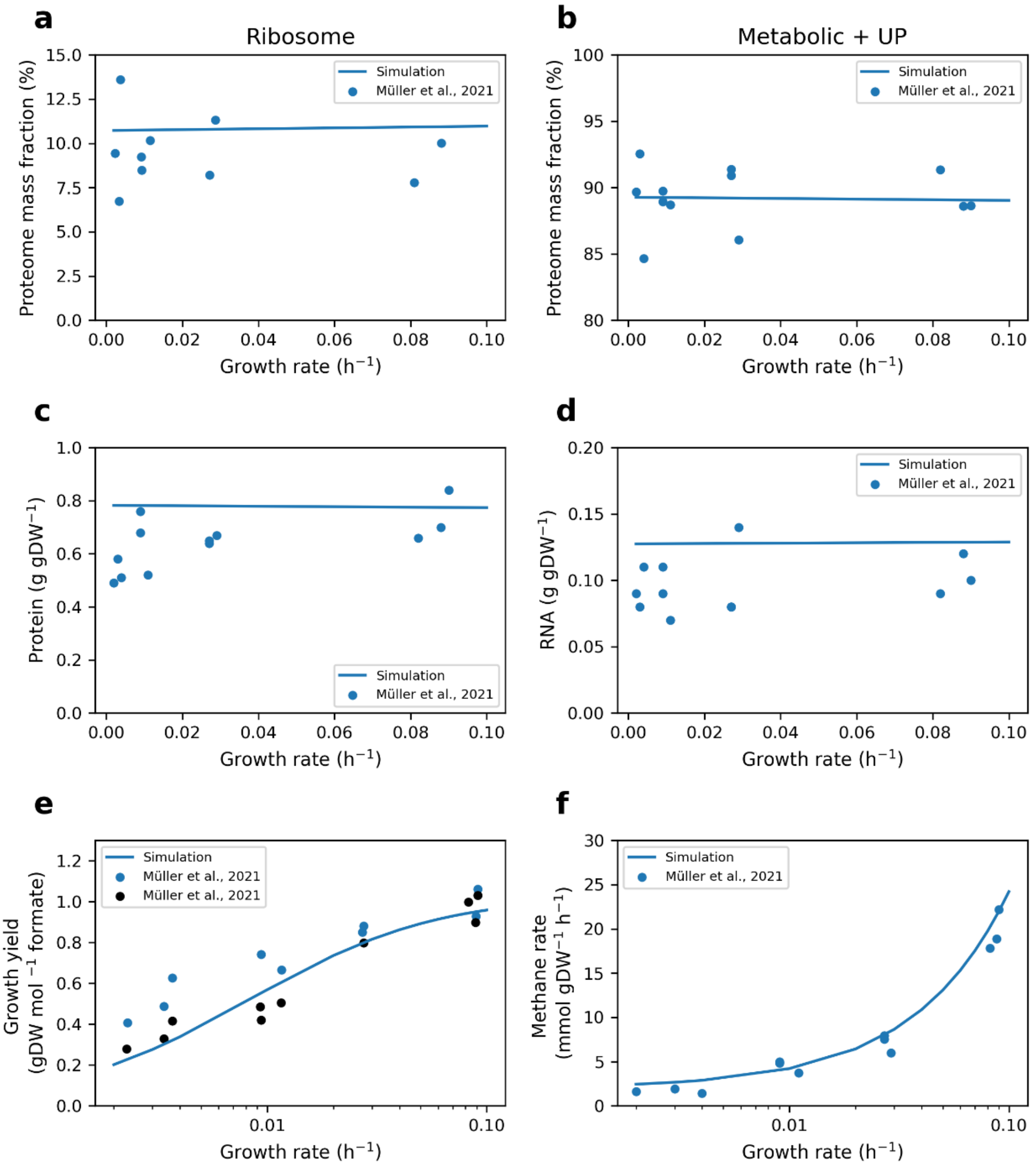

Figure S12. The model predictions under formate-limited conditions where we constrained the model to produce a glycogen mass equal to 2% of gDW. **a** Comparison between the predicted ribosomal proteome fractions and the measured data. **b** Comparison between the predicted sum of metabolic and UP proteome fractions and the measured data. **c** Comparison between the predicted protein mass and the measured data. **d** Comparison between the predicted RNA mass and the measured data. **e** Comparison of the predicted yield (gDW mol^{-1} formate) with the measured yield. Under formate-limited conditions, the black and blue symbols refer to the observed growth yield and the corrected growth yield with the cell lysis, respectively. **f** Comparison of predicted methane fluxes (mmol gDW^{-1} h^{-1}) with the measured fluxes.

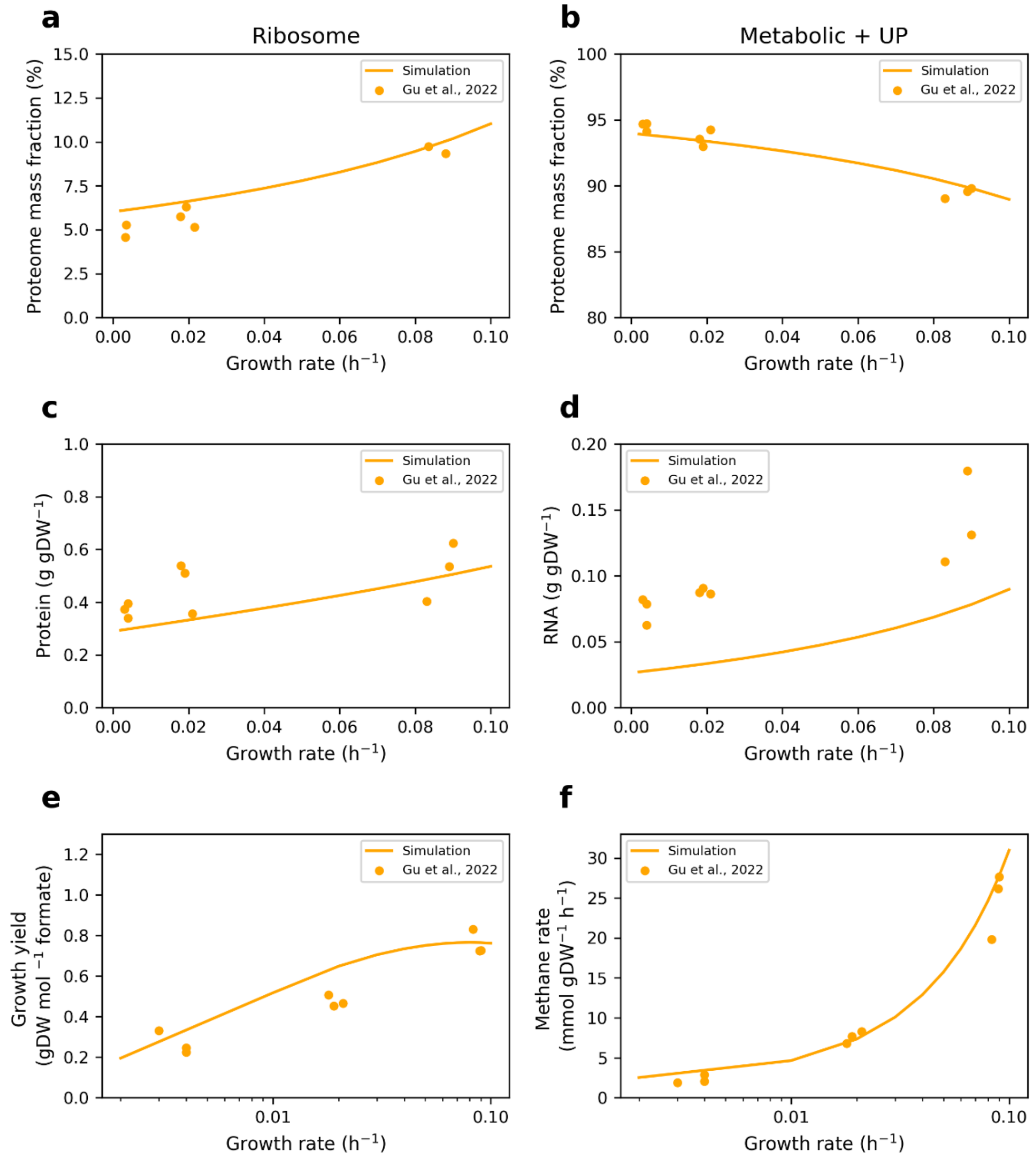

Figure S13. The model predicts under phosphate-limited conditions, where we constrained the model to free amino acid molecule instead of glycogen. **a** Comparison between the predicted ribosomal proteome fractions and the measured data. **b** Comparison between the predicted sum of metabolic and UP proteome fractions and the measured data. **c** Comparison between the predicted protein mass and the measured data. **d** Comparison between the predicted RNA mass and the measured data. **e** Comparison of predicted yield (gDW mol^{-1} formate) with the measured yield. **f** Comparison of predicted methane fluxes (mmol gDW^{-1} h^{-1}) with the measured fluxes.

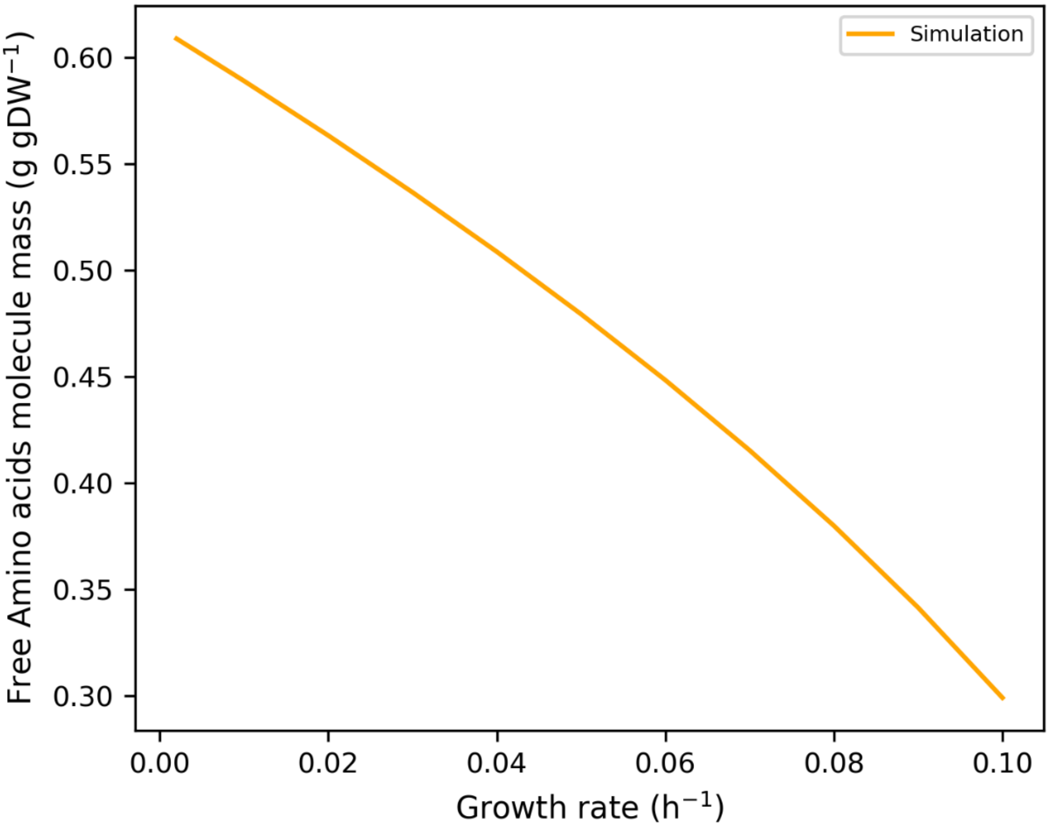

Figure S14. Model predictions of the free amino acid molecule mass under phosphate-limited conditions where we constrained the model to free amino acid molecule instead of glycogen.

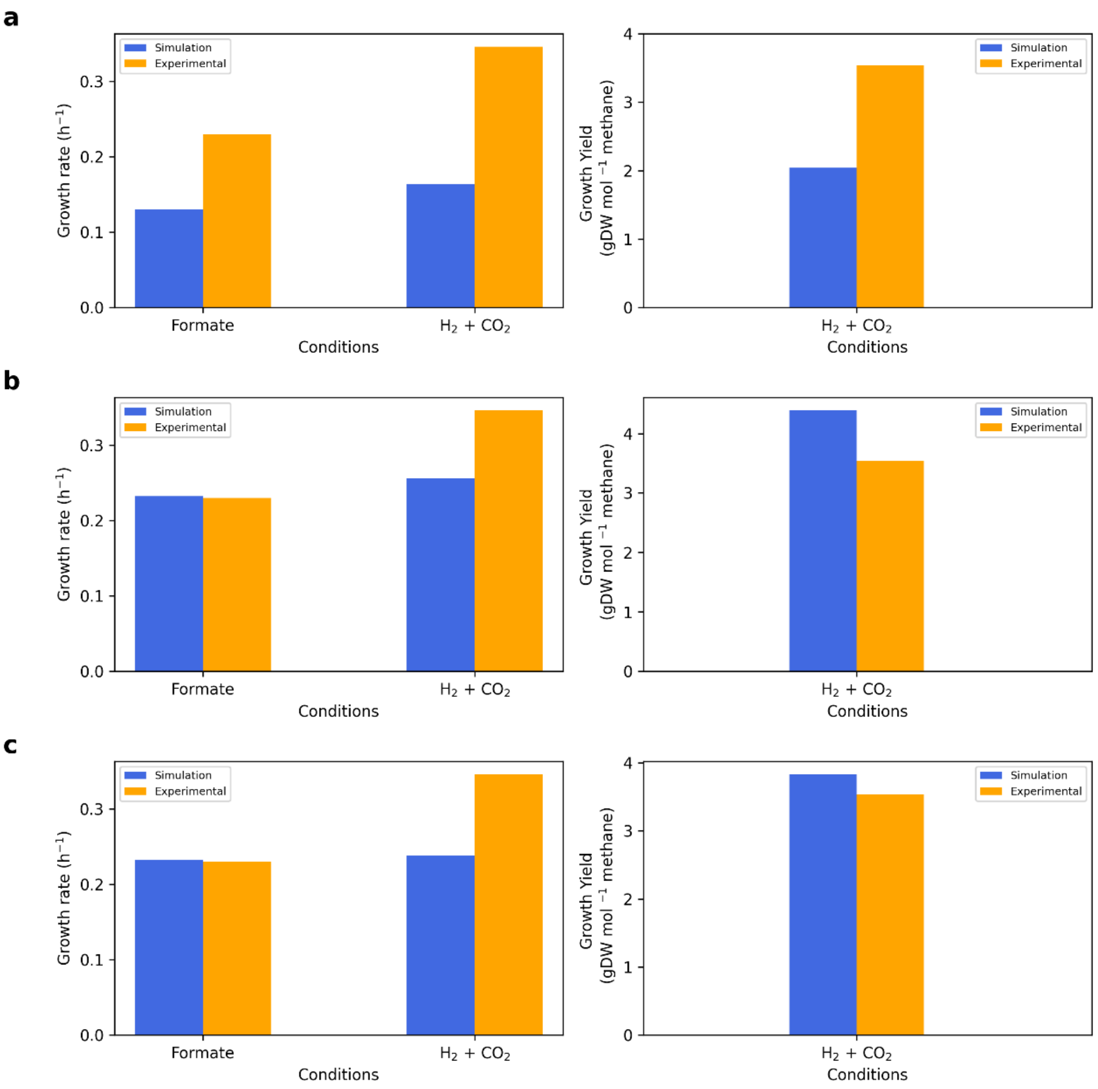

Figure S15. Effect of the UP cost ratio, GAM, and NGAM on the maximum growth rate and methane yield, under formate-limited and CO_2 with H_2 conditions. The blue and orange colors refer to the model predictions and the measured data, respectively. **a** Model predictions with values: GAM = 25, NGAM = 1, and UP = 75% under formate-limited conditions; GAM = 130, NGAM = 5, and UP = 44% under CO_2 with H_2 conditions. **b** Model predictions with values: GAM = 25, NGAM = 1, and UP = 44% for both formate-limited and CO_2 with H_2 conditions. **c** Model predictions with values: Simulation with GAM = 25, NGAM = 1, and UP = 44% under formate-limited conditions; GAM = 25, NGAM = 5, and UP = 44% under CO_2 with H_2 conditions.

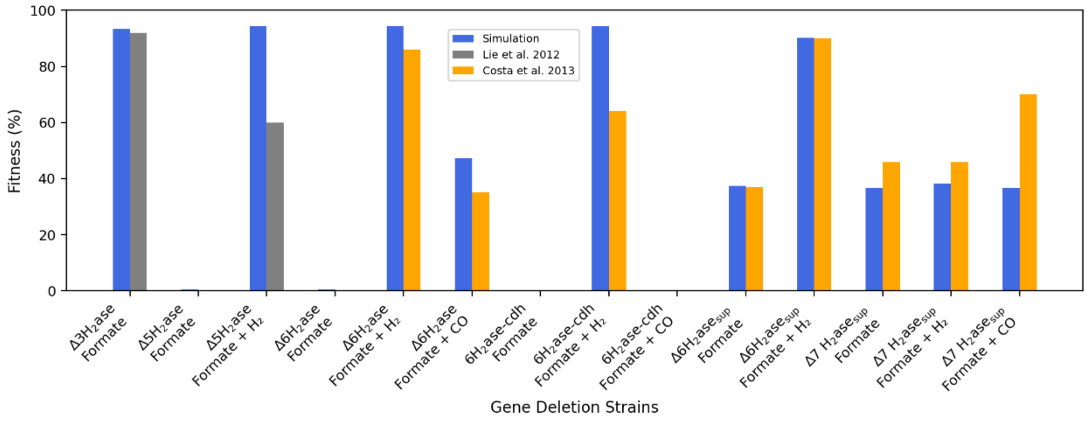

Figure S16. Comparison of the predicted and measured fitness of the different mutants when GAPOR was constrained to 1% of the total protein. The blue color represents the predicted fitness. The gray and orange colors refer to the measured fitness.

Supplementary Tables

Table S1. Mass fractions of the remaining compositions in the biomass reaction.

Composition	Metabolite	Chemical Formula	MW(g/mol)	Mass composition
0.0030965	CoA[c0]	C21H33N7O16P3S	764.51	0.002367
0.0030965	NADP[c0]	C21H26N7O17P3	741.39	0.002296
0.0030965	Mg[c0]	Mg	24.31	7.53E-05
0.0030965	NAD[c0]	C21H26N7O14P2	662.42	0.002051
0.0030965	Riboflavin[c0]	C17H20N4O6	376.36	0.001165
0.0030965	Siroheme[c0]	C42H36FeN4O16	908.6	0.002813
0.0030965	Fe2[c0]	Fe	55.85	0.000173
0.0030965	Putrescine[c0]	C4H14N2	90.17	0.000279
0.0030965	Spermidine[c0]	C7H22N3	148.27	0.000459
0.021439	dATP[c0]	C10H13N5O12P3	487.9774	0.010462
0.021439	TTP[c0]	C10H14N2O14P3	479.14	0.010272
0.0030965	K[c0]	K	39.1	0.000121
0.0030965	S-Adenosyl-L-methionine[c0]	C15H23N6O5S	399.44	0.001237
0.0030965	Co2[c0]	Co	58.93	0.000182
0.0030965	H4MPT[c0]	C30H42N6O16P	773.66	0.002396
0.0030965	CoM[c0]	C2H5O3S2	141.19	0.000437
0.0030965	FAD[c0]	C27H31N9O15P2	783.53	0.002426
0.0030965	Pyridoxal_phosphate[c0]	C8H9NO6P	246.13	0.000762
0.010608	dCTP[c0]	C9H13N3O13P3	463.9661	0.004922
0.0030965	TPP[c0]	C12H17N4O7P2S	423.3	0.001311
0.0030965	HTP[c0]	C11H20NO7PS	341.32	0.001057
0.0030965	Methanofuran[c0]	C34H40N4O15	744.7	0.002306
0.0030965	Coenzyme_F420[c0]	C29H32N5O18P	769.56	0.002383
0.010608	dGTP[c0]	C10H13N5O13P3	503.9723	0.005346
0.0030965	Cl-[c0]	Cl	35.45	0.00011
0.0030965	fe3[c0]	Fe	55.85	0.000173
0.0030965	GSH[c0]	C10H16N3O6S	306.31	0.000948
0.0030965	Ca2[c0]	Ca	40.08	0.000124
0.0030965	Calomide[c0]	C72H101CoN18O17P	1580.59	0.004894
0.0030965	ACP[c0]	C11H21N2O7PRS	356.33	0.001103
0.0030965	Factor_430[c0]	C42H46N6O13Ni	901.54	0.002792
0.0030965	Coenzyme_F420-3[c0]	C34H38N6O21P	897.67	0.00278
0.0030965	Molybdenum[c0]	Mo	95.94	0.000297
0.0030965	Archaellin[e0]			0*
0.0030965	Selenoprotein[c0]	C5H12O	88.15	0.000273
0.0030965	Saturated_CDP-archaeol[c0]	C52H99N3O13P2	1036.3	0.003209
0.0030965	Saturated_Archaetidylserine[c0]	C46H93NO8P	819.2	0.002537
-0.0030965	Dimethylbenzimidazole[c0]	C9H10N2	146.19	-0.00045
-0.0030965	apo-ACP[c0]	HOR	17.007	-5.3E-05
-0.0030965	Cobinamide[c0]	C48H73CoN11O8	991.09	-0.00307
**Mass fraction (g/gDW)**				0.072965

*Archaellin[e0] is zero because it depends on the import of the Flagellin[e0], and its molecular weight is not available.

Table S2. List of reactions that were fixed upper bound and lower bound in the growth medium.

rxn_id	reaction	lb	ub	Reference
R877	--> Nitrate[e0]	0	0	Richards et al.[4]
R881	--> L-Proline[e0]	0	0	
R883	--> Taurine[e0]	0	0	
R884	L-Glutamine[e0] -->	0	0	
R885	--> L-Glutamine[e0]	0	0	
R887	--> BET[e0]	0	0	
R889	--> HYXN[e0]	0	0	
R897	--> Cytosine[e0]	0	0	
R899	--> glycogenn-1[c0]	0	0	
R901	--> Uracil[e0]	0	0	
R907	--> Urea[e0]	0	0	
R915	--> Biomass[c0]	0	0	
R919	--> ddca[e0]	0	0	
R925	--> Spermine[e0]	0	0	
R931	--> Dephospho-CoA[e0]	0	0	
R935	--> Cobinamide[e0]	0	0	
R937	--> ocdca[e0]	0	0	
R939	--> Oxidized_glutathione[e0]	0	0	
R941	--> Myristic_acid[e0]	0	0	
R945	--> Methane[e0]	0	0	
R1256	--> Indoleacetate[e0]	0	0	
R1033	L-Alanine[e0] -->	0	0	
R1034	D-Alanine[e0] -->	0	0	
R57	ADP[c0] + H[c0] + 2.0 Phosphate[c0] --> ATP[c0] + H2O[c0] + Phosphate[e0]	0	0	
R59	ADP[c0] + H[c0] + 2.0 Phosphate[c0] --> ATP[c0] + H2O[c0] + Phosphate[e0]	0	0	
R132	3-Phosphoglycerate[c0] + 3.0 H[c0] + Reducedferredoxin[c0] --> Glyceraldehyde3-phosphate[c0] + H2O[c0] + Oxidizedferredoxin[c0]	0	0	
R433	AMP[c0] + Acetyl-CoA[c0] + PPi[c0] --> ATP[c0] + Acetate[c0] + CoA[c0] + H[c0]	0	0	
R359	16.0 ADP[c0] + H2[c0] + 6.0 H[c0] + 2.0 NH3[c0] + 4.0 Oxidizedferredoxin[c0] + 16.0 Phosphate[c0] --> 16.0 ATP[c0] + 16.0 H2O[c0] + N2[c0] + 4.0 Reducedferredoxin[c0]	0	0	
R867	Biomass reaction in the iMR539 model	0	0	In this study
R608	ATP[c0] + Pyruvate[c0] --> ADP[c0] + Phosphoenolpyruvate[c0]	0	0	Goyal et al.[5]
R1261	D-fructose-1_6-bisphosphate[c0] + H2O[c0] --> D-fructose-1-phosphate[c0] + H[c0] + Phosphate[c0]	0	0	In this study
R946	--> H2[e0]	0	0 or inf	based on growth condition
R913	--> CO2[e0]	0	0 or inf	based on growth condition
R1165	--> CO[e0]	0	0 or inf	based on growth condition
R983	--> Acetate[e0]	0	0 or inf	based on growth condition
R895	--> Formate[e0]	0	0 or inf	based on growth condition

Table S3. List of data points extracted from the exponential phase of growth curves for mutants reported by Costa et al.[11] and Lie et al.[12].

References	Conditions	Time (Hours)	OD660	Growth rate	Fitness
Costa et al.[11]	Wild-type strain (Formate)	4.030938922	0.239669427	0.353525781	1
		5.003314571	0.338842982		
		6.011048796	0.482644556		
	Δ6H_2ase (Formate + H_2)	4.013259634	0.195041314	0.304549574	0.8614635
		5.020993859	0.266115683		
		6.028729432	0.360330573		
	Δ7H_2ase$_{sup}$ (Formate)	7.266298441	0.236363629	0.162575599	0.4598691
		8.521546737	0.290909072		
		9.511601674	0.34049585		
	Δ7H_2ase$_{sup}$ (Formate + H_2)	7.266298441	0.246280959	0.163315079	0.4619609
		8.521546737	0.3041322		
		9.511601674	0.355371908		
	Δ6H_2asepGAPOR (Formate + H_2)	4.49721727	0.154340841	0.318747262	0.9016238
		6.523190971	0.288424425		
		8.059369429	0.480385861		
	Δ6H_2asepGAPOR (Formate)	12.97959176	0.229581994	0.130696567	0.3696946
		15.00556546	0.301929263		
		17.00927605	0.388745986		
	Δ7H_2ase$_{sup}$ (Formate + CO)	6.023530195	0.235200024	0.247542702	0.7002112
		7.272727273	0.332800066		
		8.539037694	0.438400047		
	Δ6H_2ase (Formate + CO)	9.001069258	0.230400036	0.12389913	0.350467
		11.02032112	0.292800064		
		13.02245963	0.379200031		
	Δ6H_2ase-Δ*cdh* (Formate + H_2)	5.012422211	0.209328393	0.227379071	0.6431754
		7.006211994	0.31119404		
		9.018633612	0.52052239		
Lie et al.[12]	Wild-type strain (Formate)	4.133408227	0.240718576	0.378033949	1
		5.159635727	0.348503069		
		6.157355217	0.517365293		
	Δ3H_2ase (Formate)	4.161916238	0.238922196	0.348014538	0.9205907
		5.159635727	0.346706621		
		6.185861053	0.4832336		
	Δ5H_2ase (Formate + H_2)	4.164759485	0.235406703	0.22860079	0.6047097
		5.180778377	0.31674641		
		6.169336336	0.372248828		

Table S4. Free amino acid molecule fraction.

Amino acid composition in the biomass reaction (mmol/gDW)	Fraction of amino acids in the free amino acid molecule (mol/mol)	Amino acid	Free amino acid molecule composition	MW of amino acids	MW of the free amino acid molecule
0.1278	0.0286566875021862	L-Methionine[c0]	0.0286566875021862 L-Methionine[c0]	149.2	4.275578
0.2467	0.0553177214928744	L-Arginine[c0]	0.0553177214928744 L-Arginine[c0]	174.2	9.636347
0.42793	0.0959550569859981	L-Alanine[c0]	0.0959550569859981 L-Alanine[c0]	89.1	8.549596
0.21107	0.0473283805249331	L-Threonine[c0]	0.0473283805249331 L-Threonine[c0]	119.1	5.63681
0.20083	0.0450322578330521	L-Aspartate[c0]	0.0450322578330521 L-Aspartate[c0]	133.1	5.993794
0.50987	0.114328523135678	Glycine[c0]	0.114328523135678 Glycine[c0]	75.1	8.586072
0.28544	0.0640044200361819	L-Lysine[c0]	0.0640044200361819 L-Lysine[c0]	146.2	9.357446
0.17946	0.0402404470981404	L-Serine[c0]	0.0402404470981404 L-Serine[c0]	105.1	4.229271
0.21909	0.0491267109925977	L-Glutamate[c0]	0.0491267109925977 L-Glutamate[c0]	147.1	7.226539
0.21909	0.0491267109925977	L-Glutamine[c0]	0.0491267109925977 L-Glutamine[c0]	146.2	7.182325
0.076146	0.0170742732906219	L-Cysteine[c0]	0.0170742732906219 L-Cysteine[c0]	121.2	2.069402
0.37539	0.0841739743462104	L-Leucine[c0]	0.0841739743462104 L-Leucine[c0]	131.2	11.04363
0.12068	0.0270601646929878	L-Tyrosine[c0]	0.0270601646929878 L-Tyrosine[c0]	181.2	4.903302
0.20083	0.0450322578330521	L-Asparagine[c0]	0.0450322578330521 L-Asparagine[c0]	132.1	5.948761
0.047202	0.0105841389943521	L-Tryptophan[c0]	0.0105841389943521 L-Tryptophan[c0]	204.2	2.161281
0.18435	0.0413369353758062	L-Proline[c0]	0.0413369353758062 L-Proline[c0]	115.1	4.757881
0.2418	0.0542189909078923	L-Isoleucine[c0]	0.0542189909078923 L-Isoleucine[c0]	131.2	7.113532
0.15452	0.0346481326513131	L-Phenylalanine[c0]	0.0346481326513131 L-Phenylalanine[c0]	165.2	5.723872
0.079264	0.0177734247118411	L-Histidine[c0]	0.0177734247118411 L-Histidine[c0]	155.2	2.758436
0.35223	0.0789807906016828	L-Valine[c0]	0.0789807906016828 L-Valine[c0]	117.1	9.248651
**The total MW of the free amino acid molecule**					126.4025

Table S5. List of maximum growth rates for each mutant under different conditions.

**conditions**	**KO Type**	μ_{max}
**Formate**	Wild Type	0.238234
	$3H_2$ase KO	0.222547
	$5H_2$ase KO	0.000961
	$6H_2$ase KO	0.000961
	$6H_2ase_{sup}$ KO (with GAPOR)	0.204898
	$6H_2$ase-Δ*cdh* KO	No growth
	$7H_2ase_{sup}$ KO (with GAPOR)	0.204898
**Formate + H_2**	$5H_2$ase KO	0.224508
	$6H_2$ase KO	0.224508
	$6H_2$ase-Δ*cdh* KO	0.224508
	$6H_2ase_{sup}$ KO (with GAPOR)	0.224508
	$7H_2ase_{sup}$ KO (with GAPOR)	0.204898
**Formate + CO**	$6H_2$ase KO	0.112734
	$6H_2$ase-Δ*cdh* KO	No growth
	$7H_2ase_{sup}$ KO (with GAPOR)	0.204898

Reference

1. Schmitt, E. et al. Recent Advances in Archaeal Translation Initiation. *Front. Microbiol.* **11**, 584152 (2020).

2. Chen, Y. et al. Proteome constraints reveal targets for improving microbial fitness in nutrient-rich environments. *Mol. Syst. Biol.* **17**, e10093 (2021).

3. Hendrickson, E. L. et al. Complete Genome Sequence of the Genetically Tractable Hydrogenotrophic Methanogen Methanococcus maripaludis. *J. Bacteriol.* **186**, 6956–6969 (2004).

4. Richards, M. A. et al. Exploring Hydrogenotrophic Methanogenesis: a Genome Scale Metabolic Reconstruction of Methanococcus maripaludis. *J. Bacteriol.* **198**, 3379–3390 (2016).

5. Goyal, N., Zhou, Z. & Karimi, I. A. Metabolic processes of Methanococcus maripaludis and potential applications. *Microb. Cell Fact.* **15**, 107 (2016).

6. Elsemman, I. E. et al. Whole-cell modeling in yeast predicts compartment-specific proteome constraints that drive metabolic strategies. *Nat. Commun.* **13**, 801 (2022).

7. Sánchez, B. J. et al. Improving the phenotype predictions of a yeast genome-scale metabolic model by incorporating enzymatic constraints. *Mol. Syst. Biol.* **13**, 935 (2017).

8. Müller, A. L. et al. An alternative resource allocation strategy in the chemolithoautotrophic archaeon Methanococcus maripaludis. *Proc. Natl Acad. Sci. USA* **118**, e2025854118 (2021).

9. von der Haar, T. A quantitative estimation of the global translational activity in logarithmically growing yeast cells. *BMC Syst. Biol.* **2**, 87 (2008).

10. O'Brien, E. J., Lerman, J. A., Chang, R. L., Hyduke, D. R. & Palsson, B. Ø. Genome-scale models of metabolism and gene expression extend and refine growth phenotype prediction. *Mol. Syst. Biol.* **9**, 693 (2013).

11. Costa, K. C. et al. H2-Independent Growth of the Hydrogenotrophic Methanogen Methanococcus maripaludis. *mBio* **4**, e00062-13 (2013).

12. Lie, T. J. et al. Essential anaplerotic role for the energy-converting hydrogenase Eha in hydrogenotrophic methanogenesis. *Proc. Natl Acad. Sci. USA* **109**, 15473–15478 (2012).
